# Molecular targets of chromatin repressive mark H3K9me3 in primate progenitor cells within adult neurogenic niches

**DOI:** 10.3389/fgene.2014.00252

**Published:** 2014-07-30

**Authors:** Michael R. Foret, Richard S. Sandstrom, Christopher T. Rhodes, Yufeng Wang, Mitchel S. Berger, Chin-Hsing Annie Lin

**Affiliations:** ^1^Department of Biology, University of Texas at San AntonioSan Antonio, TX, USA; ^2^Department of Genome Sciences, University of WashingtonSeattle, WA, USA; ^3^Department of Neurological Surgery, University of California at San FranciscoSan Francisco, CA, USA; ^4^Neurobiology, Neuroscience Institute, University of Texas at San AntonioSan Antonio, TX, USA

**Keywords:** epigenetics, histone methylation, pericentric chromatin, Papio anubis, SVZ, neurogenesis

## Abstract

Histone 3 Lysine 9 (H3K9) methylation is known to be associated with pericentric heterochromatin and important in genomic stability. In this study, we show that trimethylation at H3K9 (H3K9me3) is enriched in an adult neural stem cell niche- the subventricular zone (SVZ) on the walls of the lateral ventricle in both rodent and non-human primate baboon brain. Previous studies have shown that there is significant correlation between baboon and human regarding genomic similarity and brain structure, suggesting that findings in baboon are relevant to human. To understand the function of H3K9me3 in this adult neurogenic niche, we performed genome-wide analyses using ChIP-Seq (chromatin immunoprecipitation and deep-sequencing) and RNA-Seq for *in vivo* SVZ cells purified from baboon brain. Through integrated analyses of ChIP-Seq and RNA-Seq, we found that H3K9me3-enriched genes associated with cellular maintenance, post-transcriptional and translational modifications, signaling pathways, and DNA replication are expressed, while genes involved in axon/neuron, hepatic stellate cell, or immune-response activation are not expressed. As neurogenesis progresses in the adult SVZ, cell fate restriction is essential to direct proper lineage commitment. Our findings highlight that H3K9me3 repression in undifferentiated SVZ cells is engaged in the maintenance of cell type integrity, implicating a role for H3K9me3 as an epigenetic mechanism to control cell fate transition within this adult germinal niche.

## Introduction

Chromatin is functionally classified as euchromatin and heterochromatin, which are crucial for epigenetic controls of gene expression. Underlying the specialized chromatin structure around centromere and telomere, H3K9me3 was identified to be heterochromatin-enriched histone code to silence gene expression and prevent chromosomal instability (Czvitkovich et al., 2001; Lachner et al., 2001; Peters et al., 2001, 2002; Black et al., 2012). For instance, a previous study has shown that loss of H3K9 methylation in Drosophila causes DNA damage in heterochromatin and mitotic defect (Peng and Karpen, 2009). In mice, loss of H3K9me2/me3 causes the disruption of heterochromatin and increases telomere length (Peters et al., 2001; Garcia-Cao et al., 2004; Benetti et al., 2007). H3K9 methylation is also involved in pluripotency of embryonic stem cells (ESCs) and multipotency of neural precursor cells (NPCs), in which the pluripotent genes (e.g., Nanog, Oct4) and non-neural genes (e.g., GATA4, NODAL) gain H3K9me3 that lead to long-term repression during differentiation of human ESCs into NPCs (Golebiewska et al., 2009; Hirabayashi and Gotoh, 2010). Thus, H3K9me3 plays a repressive role in numerous neuronal and non-neuronal genes (Roopra et al., 2004; Schaefer et al., 2009) in addition to its known function in genome stability. Among all lysine methyltransferases, the KMT1 family composed of G9a/GLP and Suv39h1/h2 are characterized to be essential for H3K9me1/2 and H3K9me3 modifications, respectively (Czvitkovich et al., 2001; Lachner et al., 2001; Peters et al., 2001; Black et al., 2012). Studies from KMT1 knock-out mice demonstrated that loss of H3K9 methylation contributes to behavioral abnormalities and cognitive impairment (Schaefer et al., 2009) in addition to its protective role in genome stability. In this work, we found that H3K9me3 is enriched in the subventricular zone (SVZ), where adult neurogenesis occurs.

The SVZ is the largest neural stem cell niche, which harbors stem/progenitor cells for adult neurogenesis. The SVZ contains slowly dividing neural stem cells (NSCs) with astrocyte-like morphology. In the rodent model, NSCs give rise to transit-amplifying cells, which subsequently give rise to immature neuroblasts. These neuroblasts migrate through the rostral migratory stream (RMS) and generate interneurons in the olfactory bulb (Alvarez-Buylla and Lim, 2004; Ihrie and Alvarez-Buylla, 2011). Numerous studies have demonstrated that extracellular signals such as growth factors or morphogens have significant effects on either self-renewal of NSCs or lineage commitment (Doetsch et al., 2002; Alvarez-Buylla and Lim, 2004; Zheng et al., 2004; Jackson et al., 2006; Ihrie et al., 2011). Additionally, the intracellular effectors of adult neurogenesis include cell-cycle inhibitors (p16/INK4A and p21) (Molofsky et al., 2006), transcription factors (Doetsch et al., 2002; Shi et al., 2004; Kohwi et al., 2005; Roybon et al., 2009; Qu et al., 2010; Ihrie and Alvarez-Buylla, 2011), and epigenetic mechanisms (Ming and Song, 2011). One such epigenetic mechanism includes histone modifications (Ming and Song, 2011). In this study, we demonstrate that H3K9me3 has a distinct distribution pattern within cell populations in the rodent and non-human primate SVZ. Yet, the molecular targets of H3K9me3 in this adult germinal niche remain unknown. Therefore, we developed a technique to purify subpopulations of SVZ cells from baboon brain (*Papio anubis*) (Sandstrom et al., 2014) for genome-wide analysis by using ChIP-Seq. In addition to genes involved in cell cycle and proliferation, we found that H3K9me3 enriched for genes functioning in axon and neuron projection, cellular maintenance/organization, cell signaling, and post-translational acetylation as well. A further integrated ChIP-Seq and RNA-Seq analysis revealed that 35% of H3K9me3-enriched genes are silenced, many of which are known to function in neuronal-, hepatic-, and immunological-cell type activation. In light of previous studies showing that H3K9me3 is a chromatin repressive mark, we anticipate that H3K9me3 is critical for the maintenance of cell identity within this adult neurogenic niche through its repressive function to protect against improper lineage differentiation within the SVZ.

## Results

### H3K9me3 is expressed in germinal zones within the adult brain

**Figure 1:**
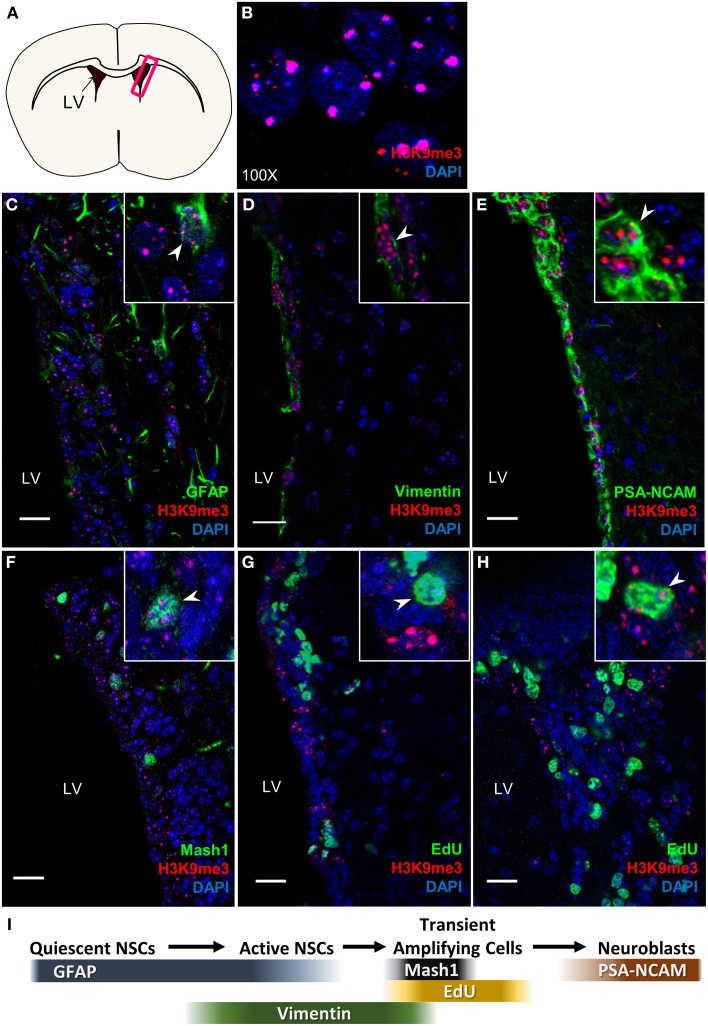
**H3K9me3 distribution in mouse SVZ. (A)** Coronal cross-section view of mouse forebrain, box indicates location of SVZ. **(B)** H3K9me3 staining pattern in SVZ cells (100X). **(C–E)** Double labeling of H3K9me3 (red) and SVZ cell-type specific markers: **(C)** NSC marker—GFAP; **(D)** Active NSC cell marker—Vimentin; **(E)** Neuroblast marker—PSA-NCAM. **(F)** Small population of H3K9me3-positive cells is colocalized with Mash1. **(G,H)** Small population of EdU^+^ cells is colocalized with H3K9me3. **(I)** Scheme displays cell type specific markers at different neural progenitor stages. LV, lateral ventricle; Images represent 12 μm coronal sections at 40X magnification; Scale bar = 20 μm.

Using co-immunostaining with antibodies specific for H3K9me3 (a pericentric chromatin staining pattern, Figure 1B) and cell type specific markers in the SVZ (Figure 1I) for the gross anatomy of 8-week (P56) old adult mouse brain (Figure 1A), we found that H3K9me3-positive cells are co-localized with GFAP which labels quiescent and active NSCs (Figure 1C), Vimentin positive active NSC (Figure 1D), and PSA-NCAM positive neuroblast populations as well (Figure 1E). As Mash1 is commonly used in the mouse to denote cells as “transient amplifying cells,” we applied co-immunostaining of Mash1 and H3K9me3 in the mouse SVZ, and found colocalization of H3K9me3 and Mash1 (Figure 1F). We then performed a 2 h EdU administration in mice to label quickly dividing transit-amplifying cells and neuroblasts, and found colocalization of EdU and H3K9me3 (Figures 1G,H). These results show that H3K9me3 is present in undifferentiated SVZ cells within rodent brain.

**Figure 2:**
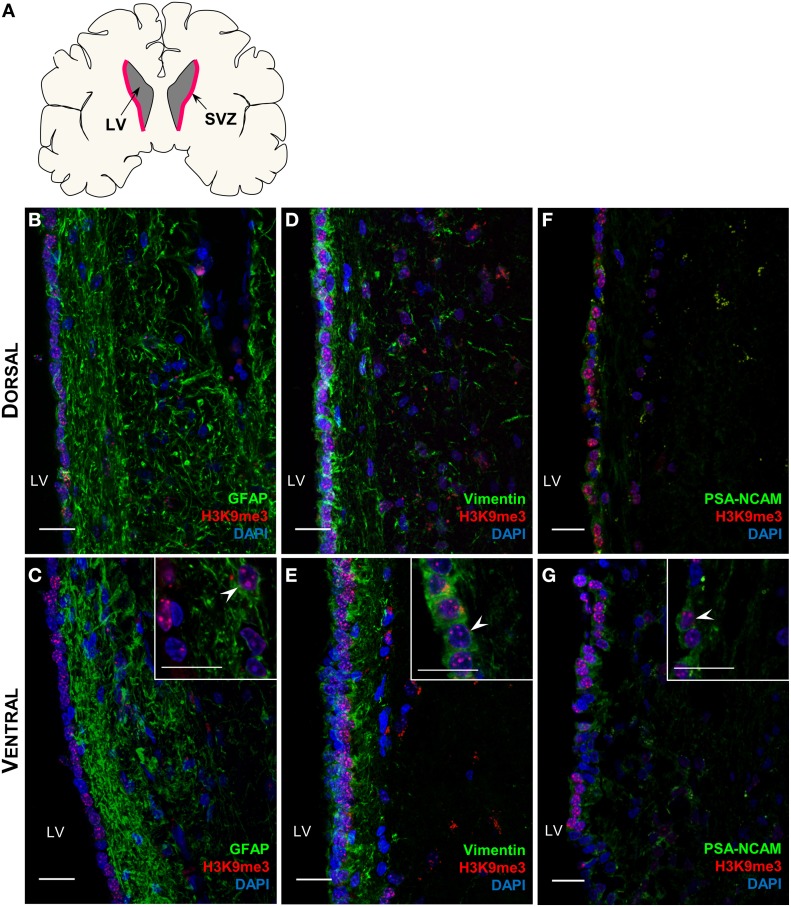
**Distribution of H3K9me3 in the neural progenitor populations of the baboon SVZ. (A)** Coronal cross-section schematic of baboon forebrain, red highlighted area annotates SVZ. **(B–G)** Double labeling of H3K9me3 (red) and neural progenitor cell specific markers (green): **(B,C)** Only a small population of H3K9me3 positive cells co-localize with GFAP (NSC marker) in dorsal and ventral SVZ. **(D,E)** An extensive population of Vimentin positive cells is co-localized with H3K9me3 along the entire SVZ. **(F,G)** Enrichment of H3K9me3 persists in PSA-NCAM (neuroblast marker) positive population throughout dorsal and ventral of SVZ. LV, lateral ventricle; Images represent 60 μm sections at 40X magnification; Inserts are 100X magnification; Scale bars = 20 μm.

The comparisons of brain volume and structure across primate species and human have shown significant correlation between baboon and human (Kochunov et al., 2010; Rogers et al., 2010), we therefore examined the H3K9me3 distribution pattern in the baboon SVZ (Figure 2A). It is worth noticing that GFAP-positive astrocytic ribbon lies in this niche and also extends toward the lateral ventricle (Figures 2B,C), resembling the previous finding in adult human brain (Sanai et al., 2004). Thus, the overall architecture of baboon SVZ geographically represents the SVZ in adult human brain (Sanai et al., 2004, 2011), corroborating the extent to which findings in baboon SVZ are relevant to human SVZ. We found that H3K9me3 is associated with GFAP- and Vimentin-positive NSCs and also PSA-NCAM-positive neuroblasts in the baboon SVZ (Figures 2D–G), suggesting that H3K9me3 has function in these cell populations.

**Figure 3:**
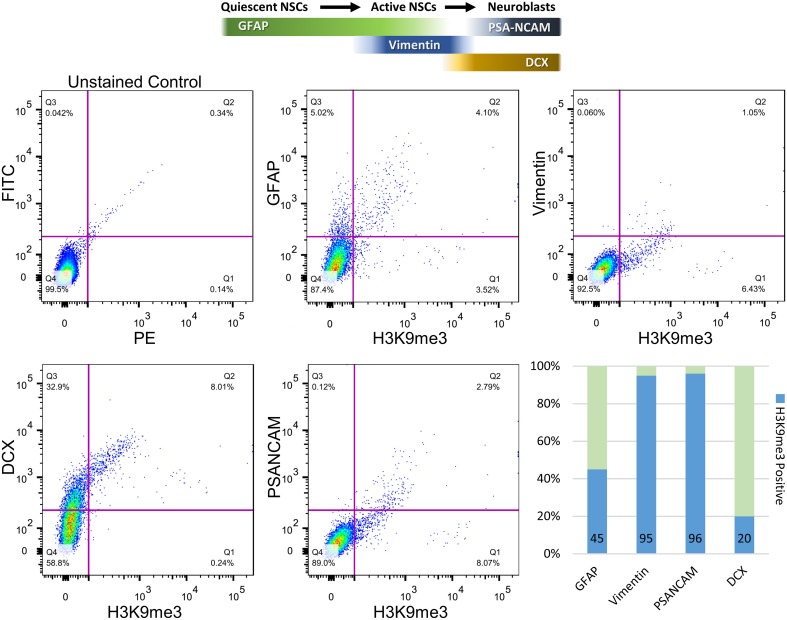
**Quantification of co-localization percentages between H3K9me3 and subpopulations of SVZ cells by flow cytometry**. After SVZ dissection, dissociated cells were analyzed by flow cytometry with antibodies against H3K9me3 and cell-type markers including GFAP (quiescent and active NSC marker), Vimentin (relatively active NSCs), PSA-NCAM (migrating neuroblast), and DCX (early and migrating neuroblast marker). Bar graph represents undifferentiated SVZ cells for each population positive for GFAP, Vimentin, PSA-NCAM, and DCX (light green). Blue annotates H3K9me3-positive percentage within each SVZ subpopulation.

To further quantify the percentages of co-localization between H3K9me3 and SVZ subpopulations, we carried out flow cytometry analysis for dissociated SVZ cells after micro-dissection of SVZ from baboon brain. Flow cytometry analysis reveals approximately 45 and 20% of GFAP- and Doublecortin (DCX)-positive populations contain H3K9me3, respectively (Figure 3). Consistent with immunostaining results, the great majority of Vimentin- and PSA-NCAM-positive cells (~95%) are colocalized with H3K9me3 (Figure 3). This quantification confirms that H3K9me3 is enriched in undifferentiated SVZ cells, while the abundance of H3K9me3 varies across different cell populations within the SVZ.

### H3K9me3 epigenetic landscape in the SVZ cells

**Figure 4:**
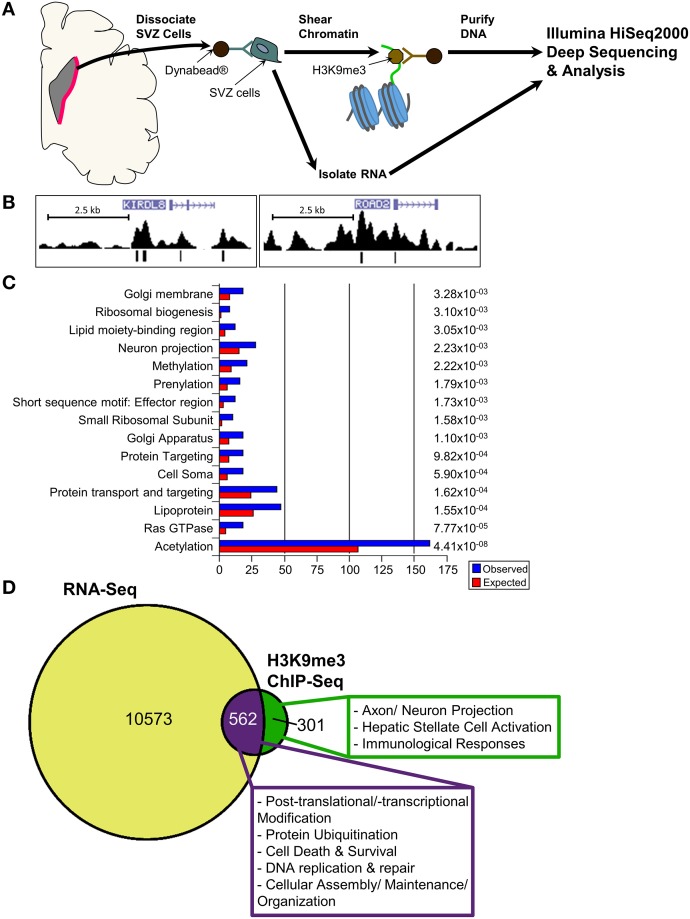
**ChIP-Seq and RNA-Seq analyses for *in vivo* SVZ cells from baboon brain. (A)** Scheme displays purification of *in vivo* baboon SVZ cells and processing for ChIP-Seq with H3K9me3 antibody and for RNA-Seq. **(B)** Peak views of representative H3K9me3 enriched genes KIRDL8 and ROAD2. [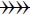] within the genes indicate the transcript direction and strand sense. H3K9me3 signal is “mapped read density” after normalization to unmodified H3—vertical bars in peak track annotate significant reads within the density plot. The H3K9me3 peaks were called at FDR0.05, which is derived from a comparison of mapped read enrichment relative to a local background model based on the binomial distribution. **(C)** Top significant GO list for H3K9me3-enriched genes, *p*-value shown at right side. **(D)** RNA-Seq analysis uncovered 11099 genes in the undifferentiated SVZ cells are detectable at transcriptional level. While 562 detectable genes are enriched with H3K9me3 (purple portion), 301 of H3K9me3-enriched genes are not detectable (green portion). For characterization of these detectable or undetectable H3K9me3-enriched genes in baboon SVZ cells, IPA prediction reveals the top canonical networks and pathways under H3K9me3 regulation in the undifferentiated SVZ cells as shown in the box.

**Figure 5:**
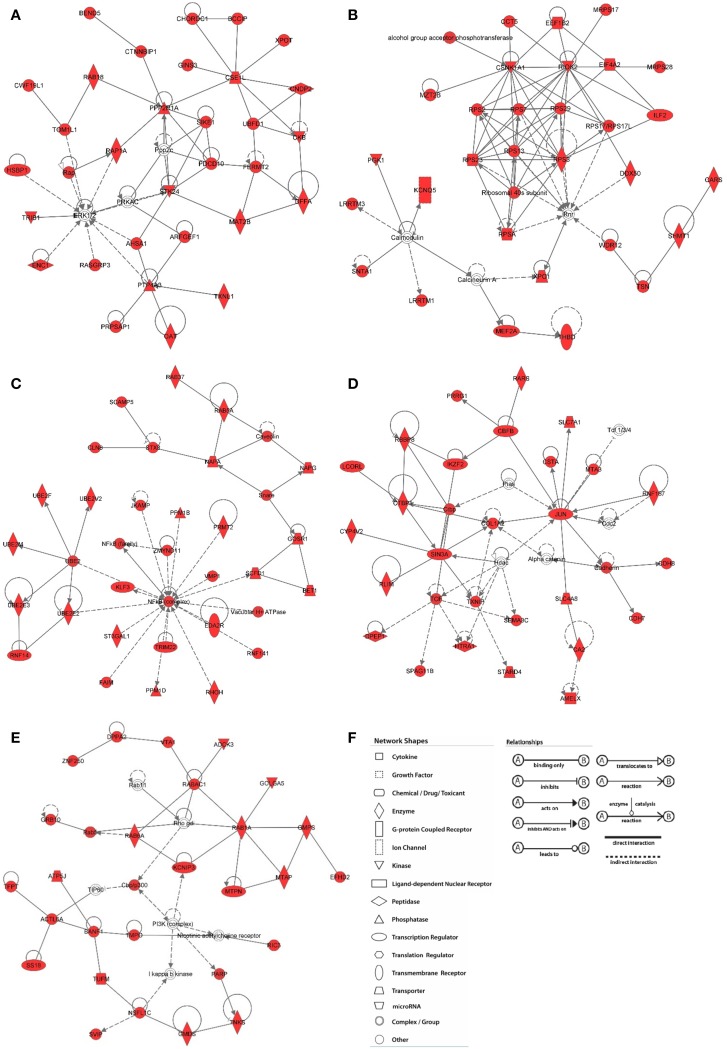
**Network analysis of H3K9me3 enriched genes in baboon SVZ cells**. Top networks were predicted by Ingenuity Pathway Analysis software. The shaded focus genes (red highlight) were enriched with H3K9me3 identified by ChIP-Seq analysis. Node shape reflects the role of each element in the network and the direction and arrowhead shapes of each edge represent different types of interactions (key at panel **F)**. **(A)** Cell morphology and organization; **(B)** RNA post-transcriptional modification; **(C)** Post-translational modifications; **(D)** Cell cycle; **(E)** Cellular growth and proliferation.

**Figure 6:**
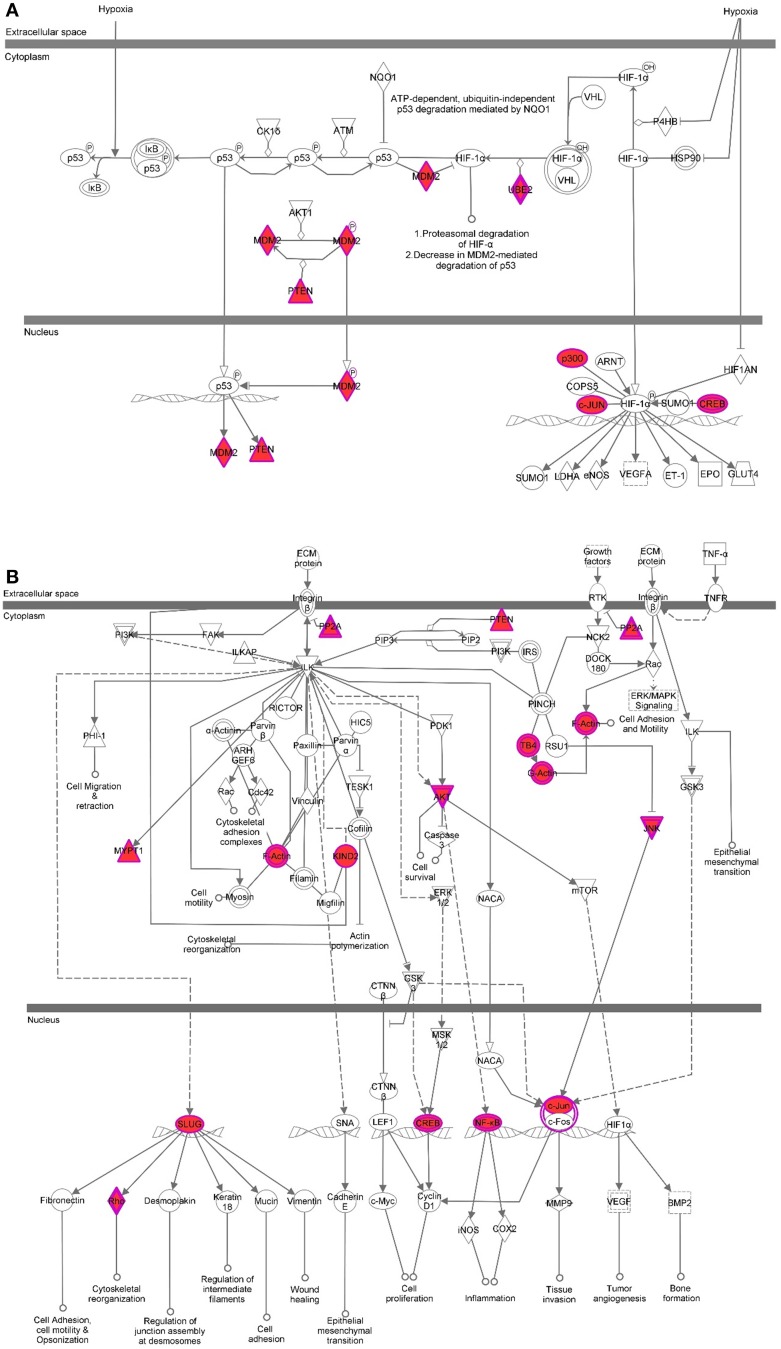
**Pathway analysis for H3K9me3 enriched genes in baboon SVZ cells**. Top canonical pathways were predicted by Ingenuity Pathway Analysis software. The shaded focus genes (red highlight) were enriched with H3K9me3 identified by ChIP-Seq analysis. The node shape reflects the role of each element in the pathway and the direction and arrowhead shapes of each edge represent different types of interactions (see the key at Figure 5F). Representative signaling pathways involve AKT, c-JUN, CREB, MDM2, p300, PTEN, PP2A induced by either hypoxia or growth factors are illustrated **(A,B)**.

While results from our study highlight the distinct localization pattern of H3K9me3 in the SVZ of rodent and non-human primate, the molecular targets of H3K9me3 in this germinal niche remain to be determined. Currently, most of the genome-wide associated studies in neural stem/progenitor cells are conducted in culture to obtain sufficient materials for analyses. However, the *in vitro* cell culture system cannot fully recapitulate *in vivo* epigenetic landscape since the metabolites yielded from cultured condition including acetyl and methyl donors can alter the status of histone acetylation and methylation (Black et al., 2012). To ascertain characteristics of SVZ cells as they exist *in vivo* for identification of the genomic loci that carry the H3K9me3 modification, we developed a technique to purify SVZ cells directly from the baboon brain within a short post-mortem interval (<20 min). We utilized conjugated Dynabeads with antibodies against SVZ cell type-specific markers to purify dissociated cells following dissection of the SVZ (Figure 4A) (Sandstrom et al., 2014). This technical innovation preserves the nature of distinct SVZ cell types and is ideal for genome-wide analysis to uncover H3K9me3 enriched loci in the SVZ through ChIP-Seq with antibodies specific for H3K9me3. Additionally, this approach ensures that H3K9me3 positive cells from the adjacent striatum are excluded from the genome-wide analysis of the SVZ cells. DNA obtained from each ChIP pull-down was sequenced to high depth (200 million tags; 36 bases) by using Illumina HiSeq2000 sequencer (Figure 4A). We did additional runs of ChIP-Seq as replicates with different vendor's antibody H3K9me3 and independent sample preparation. This approach yielded a confident list of H3K9me3 enriched loci across independent sets of deep-Seq. Because the baboon gene annotation is not currently available, we alternatively compiled, processed, and aligned the sequence reads to the Jan. 2006 rhesus macaque (*Macaca mulatta*) draft assembly, Mmul_051212 (Gibbs et al., 2007) (http://www.ncbi.nlm.nih.gov/genome/assembly/237568/) and the UCSC genome browser version: rheMac2 (http://hgdownload.cse.ucsc.edu/downloads.html#rhesus). Of note, there is only 2% difference at the genomic level between baboon and rhesus macaque, thus, we were able to identify 863 unique H3K9me3-enriched genes from independent sets of ChIP-Seq (FDR = 0.05) by using the MACS2 and closest-features program from the BEDOPS tool set (Neph et al., 2012) (Figure 4B; Supplemental Table 1i). Gene ontology with functional annotation analysis reveals that molecular functions associated with these genes include acetylation, axon/neuron projection, and protein targeting/import (Figure 4C; Supplemental Table 1ii) with connection to neurological disorders. While imprinted genes are known to be involved in broad aspects of biology including brain function, imprinting dysregulation has been associated with several neurodevelopmental and neurological disorders. To elucidate whether there are imprinted genes among H3K9me3 targets in the SVZ cells, we undertook an overlap comparison between the lists of identified imprinted genes (Luedi et al., 2005) and H3K9me3 targets identified from our ChIP-Seq analysis. We found a total of 11 genes that are imprinted and enriched with H3K9me3. These genes have known functions in CNS fate commitment, glial differentiation, neural projection, and neurite outgrowth, suggesting a role of H3K9me3 in collaboration with imprinting mechanism to maintain the populations of undifferentiated SVZ cells. Among the H3K9me3-enriched genes (*n* = 863), the top biological networks predicted by Ingenuity Pathways Analysis (IPA) are involved in (1) cell morphology and cellular assembly; (2) post-transcriptional and post-translational modifications; (3) protein synthesis; (4) cell cycle; and (5) cellular growth and proliferation (Figure 5; Supplemental Table 1iv). Additionally, the top biological pathways by IPA prediction include protein ubiquitination as well as signaling pathways involving AKT, BAX, c-JUN, MDM2, p300, P53, PP2A, and PTEN (Figure 6; Supplemental Figures 1–3).

To further characterize the functional consequence of H3K9me3 enrichment on target genes, we performed RNA-Seq for purified baboon SVZ cells and applied integrated analysis between ChIP-Seq and RNA-Seq. We identified 562 genes enriched with H3K9me3 are detectable by RNA-Seq, which are associated with cellular assembly/maintenance/organization, post-transcriptional and translational modifications, signaling pathways, and DNA replication. We also found that 301 of H3K9me3-enriched genes involved in axon/neuron or hepatic stellate cell activation and immunological response are not detectable by RNA-Seq (*p*-value = 2.2e-16) (Figure 4D; Supplemental Table 1iii). Given that H3K9me3 can act as short-term and long-term repression, we anticipate 35% (301/863) of H3K9me3-enriched genes to be under long-term repression. Lastly, H3K9me3-enriched genes in the acetylation category from our GO analysis (Figure 4C) were previously characterized with roles in histone acetylation that regulate gene expression or in protein acetylation that regulates protein stability, localization, and interactions with other molecules. Numerous H3K9me3-enriched genes associated with cellular assembly, maintenance, organization, and signaling are known to be regulated by histone acetylation (Cho and Cavalli, 2014). In addition to histone acetylation, a set of H3K9me3-enriched genes with connection to neurological disorders linked to basal ganglia malfunction are known to have post-translational acetylation on the protein as well (Lopez-Atalaya et al., 2013; Valor et al., 2013a,b). To further explore the relationship between H3K9me3 enrichment and the set of 162 H3K9me3-enriched genes in the acetylation category, we carried out an integrated analysis and found that 138 of H3K9me3-enriched genes in acetylation category are detectable by RNA-Seq, whereas 24 genes are not detectable by RNA-Seq (Supplemental Figure 4). IPA analysis reveals that the 24 undetectable genes are involved in protein degradation and endocrine system development, while the 138 detectable genes are associated with signaling, nervous system development and function, as well as cellular assembly, maintenance, morphology, and organization. Since histone acetylation is highly associated with activation of gene expression and protein acetylation is critical for stability and localization of protein, we anticipate that the interplay between H3K9me3 and acetylation on common sets of genes may modulate the balance of long- and short-term repression.

## Discussion

Negative regulation of transcription is important in establishing and maintaining cell-type specific gene expression patterns. One such negative regulation can be achieved by H3K9me3 epigenetic repression. During development, many neuronal genes are subject to repression outside the nervous system to maintain neuronal specificity. For instance, neuronal genes in terminally differentiated fibroblasts are silenced. In this regard, the chromatin repressive mark H3K9me3 was identified to participate in either short- or long-term repression. Although epigenetic mechanisms controlling cell fate specification have been intensively studied in the developing embryonic central nervous system (Lim et al., 2009; Hirabayashi and Gotoh, 2010), whether the specification or maintenance of multipotency of neural progenitors in the adult brain relies on similar epigenetic regulations as in developing brains remains unknown. In this study, we demonstrate that H3K9me3 is associated with NSCs and is persistently enriched in the neuroblast population in both the adult rodent and baboon SVZ. Given the structural and genomic correlations between baboon and human, findings in baboon are more relevant to human with regards to the molecular targets of H3K9me3 in this germinal niche.

To uncover the molecular targets of H3K9me3 in adult baboon SVZ, we developed a technique to overcome the spatial complexity of the SVZ and to purify undifferentiated SVZ cells for genome-wide analysis. Our approach will be of considerable interest to those applying the genome-wide cutting edge techniques for cell lineage study. We identified that H3K9me3 is enriched for genes involved in the network of cell cycle and the signaling pathways of PTEN, MDM2, and AKT. These findings implicate that epigenetic regulation through H3K9me3 is associated with cellular growth and proliferation. Intriguingly, significant sets of H3K9me3-enriched genes with identified functions in axon/neuron projections and hepatic or immunological activity are not expressed. As mature axon/neuron and non-neuronal genes should be silenced in this germinal niche, we reason that the repression through H3K9me3 is a mechanism to secure the identity of undifferentiated SVZ cells. In summary, as neurogenesis proceeds, numerous genes must go through active, poised, and repressed states to coordinate lineage commitment. The extent of neurogenesis can be inferred from integrated regulations through signaling pathways and different genetic/epigenetic mechanisms. Our findings suggest that H3K9me3 regulates adult neurogenesis, at least in part, by suppressing a subset of genes to maintain SVZ niche properties and to tightly regulate lineage specification in order to coordinate proper timing for adult neurogenesis.

## Materials and methods

All animal experiments were approved by the guidelines of the Institutional Animal Care and Use Committee of the University of Texas at San Antonio (UTSA) and Texas Biomedical Research Institute/Southwest National Primate Research Center (SNPRC) at San Antonio.

### Immunostaining and confocal imaging

#### Mouse

Mice were trans-cardially perfused and fixed using 1X PBS^(−)^ and 4% paraformaldehyde (PFA), respectively. Brains were cryoprotected in 30% sucrose prior to OCT embedding. For co-localization staining, 12 μm frozen sections were processed for immunostaining with antibodies against H3K9me3 (Upstate #07-422; 1:500), Glial Fibrillary Acidic Protein (GFAP)—clone GA5 (Millipore #MAB3402; 1:500), Vimentin (Sigma V2258; 1:500), Polysialic Acid-NCAM (PSANCAM)—clone 2-2b (Millipore #MAB5324; 1:500), Mash1(Abcam #ab38556; 1:500), and EdU 5-ethynyl-2′-deoxyuridine (Life Technology, #C10337 Click-iT® EdU).

#### Baboon

Coronal slices of fresh baboon forebrain were taken to obtain the SVZ and adjacent brain regions, which were subsequently, fixed in 4% PFA overnight and then cryoprotected in 30% sucrose before OCT embedding. For co-localization staining, 60 μm floating sections were processed for immunostaining with the antibodies listed above.

Vectashield with DAPI (Vector Laboratories Ltd # H-1200) was used for mounting medium and nuclear counter stain. Secondary antibodies AlexaFIuor 488 (Molecular Probes, 1:1000) and AlexaFluor 594 (Molecular Probes 1:1000) were utilized for fluorescent labeling. Mouse SVZ images were acquired under a Zeiss510 confocal microscope (40X and 100X oil immersion objectives). Baboon SVZ images were taken under a Zeiss710 two-photon confocal microscope (40X and 100X oil immersion objectives). Z-stacks were projected to single plane using Zen 2012 (Carl Zeiss Microscopy; black edition). All images were analyzed using ImageJ (NIH; version 1.47).

### Cell types purification for ChIP-Seq analysis

Antibody against SVZ cell type markers, such as GFAP, Vimentin, PSA-NCAM, or Doublecortin was manually conjugated to Dynabeads (Dynabeads®-Protein A, Life Technology). We then used the Dynabeads-conjugated antibody to purify cells immediately dissociated from SVZ microdissection. Briefly, cells from fresh dissected baboon SVZ were immediately dissociated with Accutase, subsequently equilibrated in binding buffer containing phosphate-buffered saline (PBS), saponin, 1X protease inhibitor cocktail (Roche), and subjected to Dynabeads-conjugated antibody purification. The purified cells were crosslinked in 1.1% formaldehyde before chromatin shearing by Diagenode Bioruptor. The resulting sheared chromatin fragments in a size range between 200–500 base pairs were then incubated with H3K9me3 antibody-conjugated Protein A Dynabeads overnight. Additional runs of ChIP-Seq were performed as replicates. Each replicate included independent sample preparation and a different vendor H3K9me3 antibody to ensure no bias was introduced through a specific antibody (Millipore/Upstate #07-442; Active Motif #39162; Life Technology Dynabeads protein A).

For normalization, the aliquot of sheared chromatin fragments were incubated with antibody against total histone 3- conjugated Protein A Dynabeads (unmodified H3 antibody, Millipore #05-499; 1:1000). Subsequently, enriched chromatin fragments were eluted, de-crosslinked purified for library preparation (Illumina Library Kit), and sequenced with 200 million tags through Illumina HiSeq2000 sequencer. 56 ng of H3K9me3 ChIP-DNA were applied for library preparation and 5pM of libraries were loaded into Illumina sequencer. The resulting 121,743,940 pass filter reads were aligned for peak calls.

### Sequence alignment and peak calling

The Rhesus macaque (rheMac2) gene annotation derived from the NCBI RefSeq project (http://nar.oxfordjournals.org/content/33/suppl_1/D501.full) was constructed and is maintained at the UCSC genome browser. Alignments were generated by using the Bowtie alignment program version 0.12.7., with a maximum of 2 mis-matches allowed in the mapping reads. Aligned read enrichments were detected using the MACS2 peak finding program. Peak calls were generated by normalizing to unmodified H3 DNA, and using an FDR of 0.05. ChIP-Sequencing raw data and processed peak calls have been deposited to GEO under the accession ID GSE59074.

### Go, network, and pathway analysis

Duplicate gene references were removed prior to GO, network, and pathway analyses. DAVID Functional Annotation Tool (DAVID Bioinformatics Resources 6.7, NIAID/NIH) was utilized to perform GO analysis and significance cutoff was set at a *p*-value of <0.05. Network and canonical pathway analyses were performed using Ingenuity Pathway Analysis (IPA) (Ingenuity® Systems, Redwood City, CA, USA). The top 5 networks and pathways presented in this paper were determined by IPA (*p*-value < 0.003 and Fischer's Test Score > 34, respectively).

### RNA-Seq analysis

Total RNA was extracted from purified baboon SVZ cells using TRIzol reagent and sequencing libraries were generated with Illumina RNA-Seq library kit. Paired-end RNA-deepSeq (76 base pair; >300 million tag reads; 269,081,636 mapped reads) were aligned to hg19. DESeq was used to normalize raw read counts; and Cufflink reports read counts and estimated FPKM (fragments per kilobase of exon per million fragments mapped; http://cufflinks.cbcb.umd.edu/faq.html#fpkm). Genes with expression values >1 FPKM were considered for subsequent analyses. RNA-Sequencing data have been deposited to GEO under the accession ID GSE58527.

### Flow cytometry

Disassociated SVZ cells were incubated on ice for 10 min with anti-mouse CD16/CD32 (clone 2.4G2; BD Pharmingen) to block FcRs. Cells were then incubated on ice for 1 h with primary antibodies (anti-H3K9me3, GFAP, Vimentin, PSA-NCAM, and Doublecortin), and subsequently labeled with Alexa Fluor 488- and PE-conjugated secondary antibodies (BD Biosciences). Primary antibody resources: H3K9me3 (Millipore/Upstate #07-442; 1:500); Doublecortin (Millipore clone2G5 #MABN707; 1:250); Polysialic Acid-NCAM, clone 2-2b (Millipore #MAB5324, Lot# 1966892; 1:250); Glial Fibrillary Acidic Protein, clone GA5 (Millipore #MAB3402, Lot#1993774; 1:250); Vimentin (Sigma V2258; 1:250); For analysis, controls including the positive controls stained with each antibody separately, isotype controls, and the unstained cells were used for gate compensation. Flow data was acquired on a LSR-II flow cytometer (BD Biosciences) configured with an argon 488 laser with a 505 LP dichroic and 525/50 filter to detect Alexa fluor 488 and a green 510 laser with a 735 LP dichroic and a 575/26 filter to detect PE. Compensation and data analysis was performed using FlowJo software (Tree Star, Inc, Ashland, OR).

### Conflict of interest statement

The Guest Associate Editor Michael T. Chin declares that, despite being affiliated to the same Institution as the author Richard S. Sandstrom, the review process was handled objectively. The authors declare that the research was conducted in the absence of any commercial or financial relationships that could be construed as a potential conflict of interest.

## Supplementary material

Supplemental Figure 1 **Protein Ubiquitination Pathway**. One of top four canonical pathways predicted by Ingenuity Pathway Analysis for H3K9me3 enriched genes in baboon SVZ cells. The shaded focus genes (red highlight) were enriched with H3K9me3 identified by ChIP-Seq analysis. The node shape reflects the role of each element in the pathway and the direction and arrowhead shapes of each edge represent different types of interactions (see key at bottom-left panel).

Supplemental Figure 2 **P53 signaling pathway**. One of top four canonical pathways predicted by Ingenuity Pathway Analysis for H3K9me3 enriched genes in baboon SVZ cells. The shaded focus genes (red highlight) were enriched with H3K9me3 identified by ChIP-Seq analysis. The node shape reflects the role of each element in the pathway and the direction and arrowhead shapes of each edge represent different types of interactions (see key in Supplemental Figure 1).

Supplemental Figure 3 **Hepatic stellate cell activation pathway**. One of top four canonical pathways predicted by Ingenuity Pathway Analysis for H3K9me3 enriched genes in baboon SVZ cells. The shaded focus genes (red highlight) were enriched with H3K9me3 identified by ChIP-Seq analysis. The node shape reflects the role of each element in the pathway and the direction and arrowhead shapes of each edge represent different types of interactions (see key in Supplemental Figure 1).

Supplemental Figure 4 **The integrated analysis between RNA-Seq and the set of H3K9me3-enriched genes in the acetylation category from our GO analysis**. 138 of H3K9me3-enriched genes in acetylation category are detectable by RNA-Seq, whereas 24 genes are not detectable by RNA-Seq. For characterization of these detectable or undetectable H3K9me3-enriched genes in baboon SVZ cells, IPA prediction reveals the top canonical networks and pathways as shown in the box.

Supplemental Table 1 **(i)** Summary of Enriched Loci of H3K9me3; **(ii)** Gene Ontology by DAVID for H3K9me3-enriched genes in Baboon SVZ Cells; **(iii)** Integrated analyses for ChIP-Seq and RNA-Seq; **(iv)** A subset of H3K9me3-enriched genes was characterized as imprinting genes.
