# Supplementary figures and images for: Molecular targets of chromatin repressive mark H3K9me3 in primate progenitor cells within adult neurogenic niches
Source: Front Genet. 2014 Jul 30;5:252. doi: 10.3389/fgene.2014.00252 (PMC4115620; doi:10.3389/fgene.2014.00252)

Supplemental Figure 1

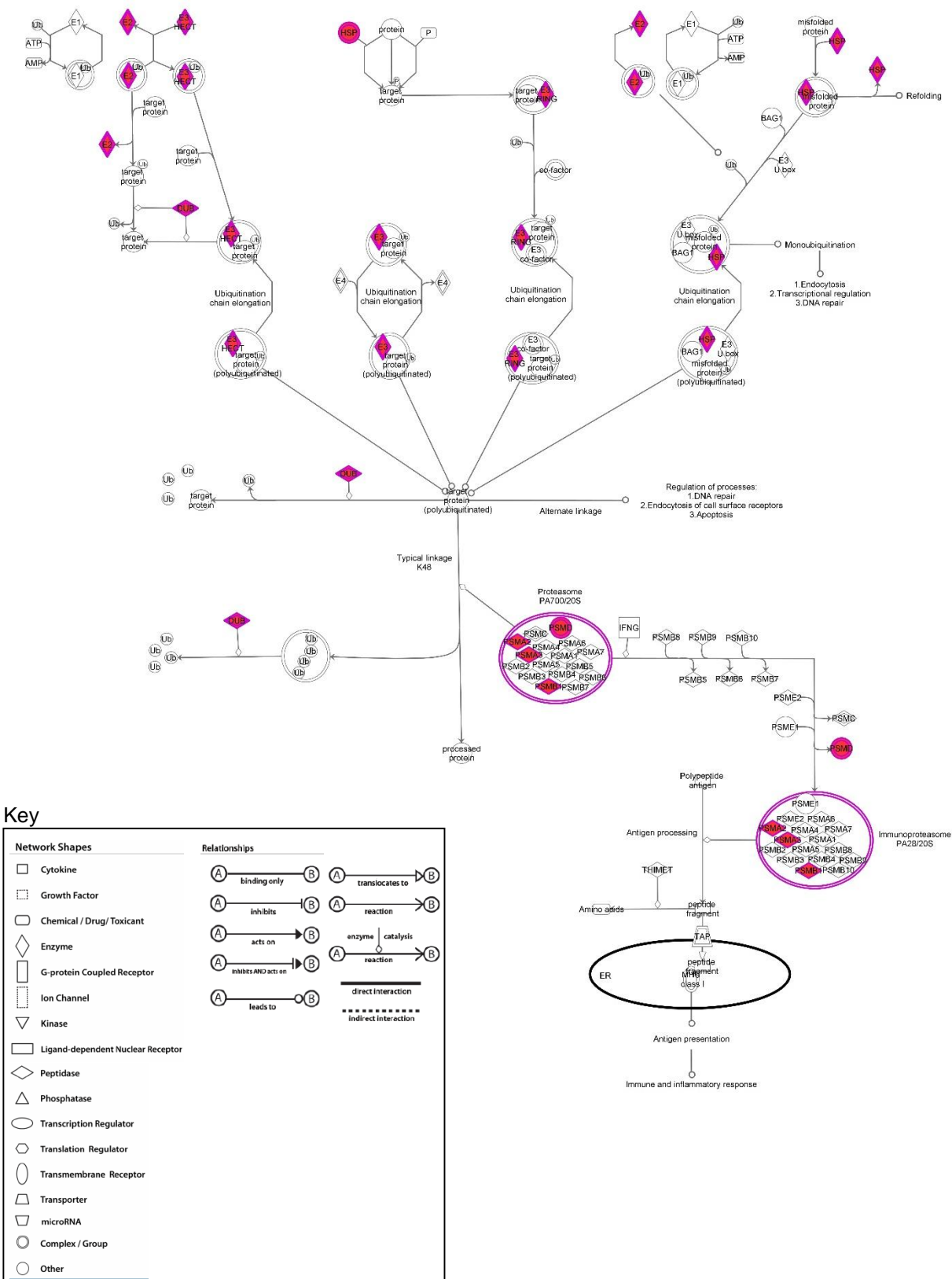

## Supplemental Figure 2

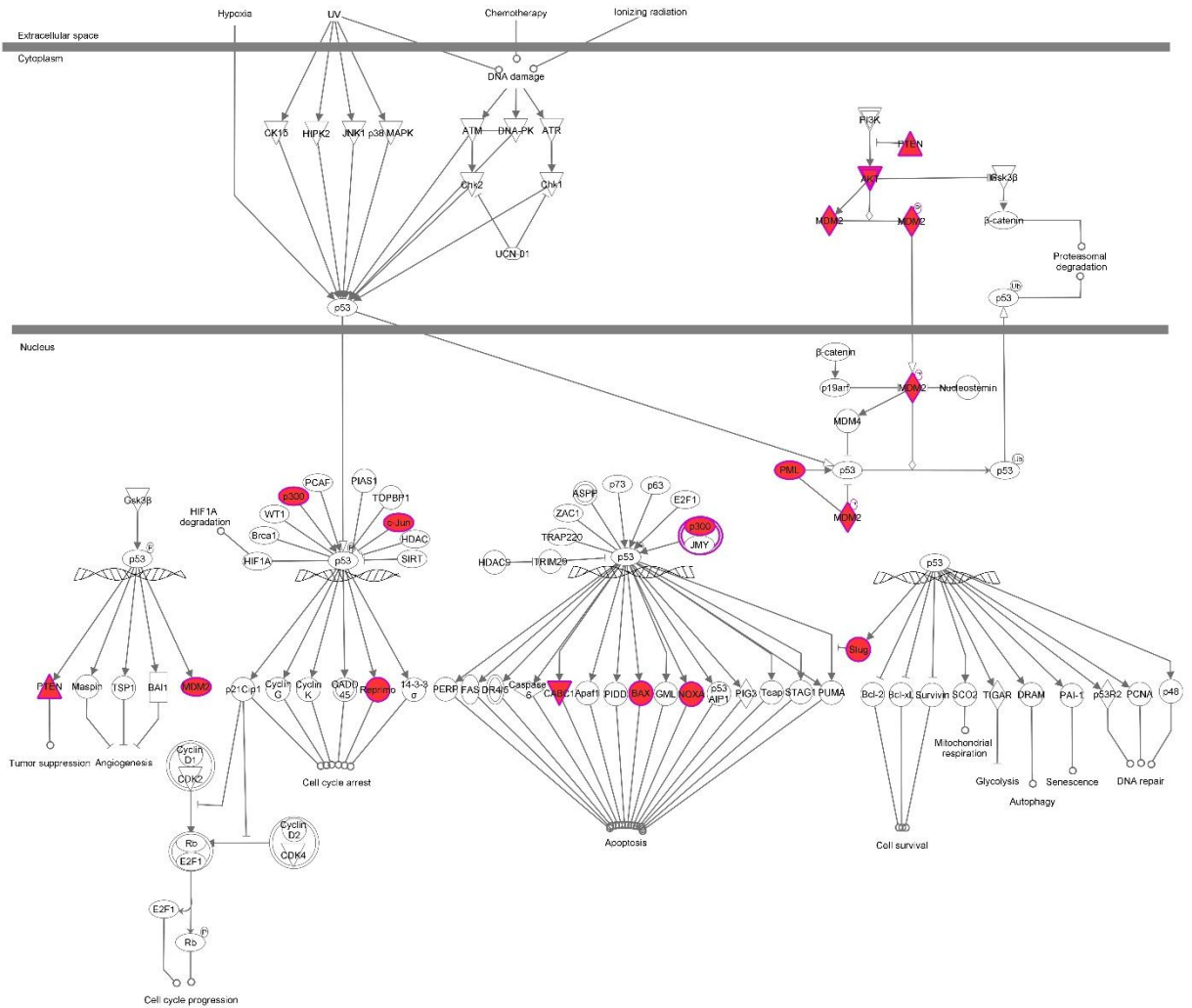

Supplemental Figure 3

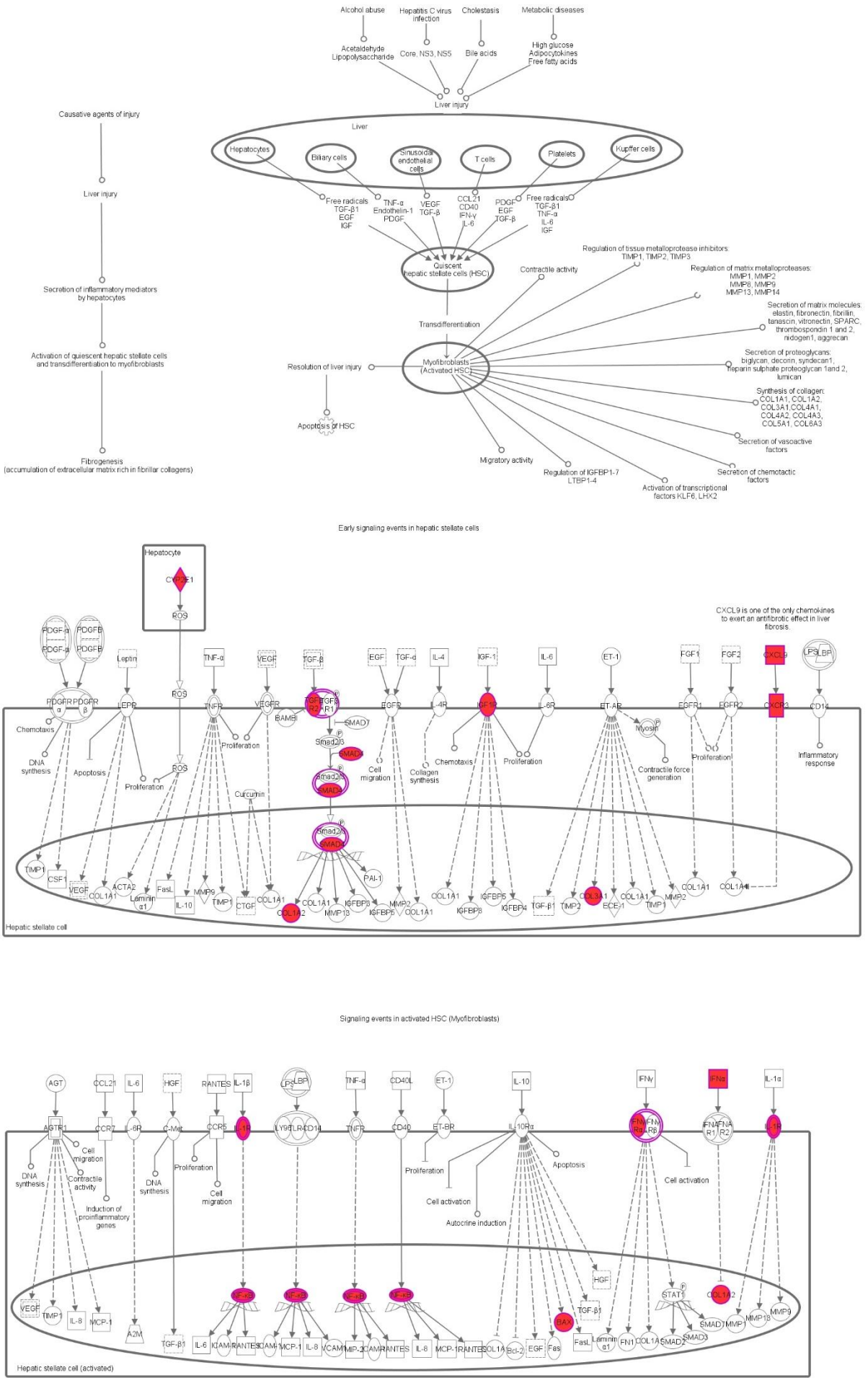

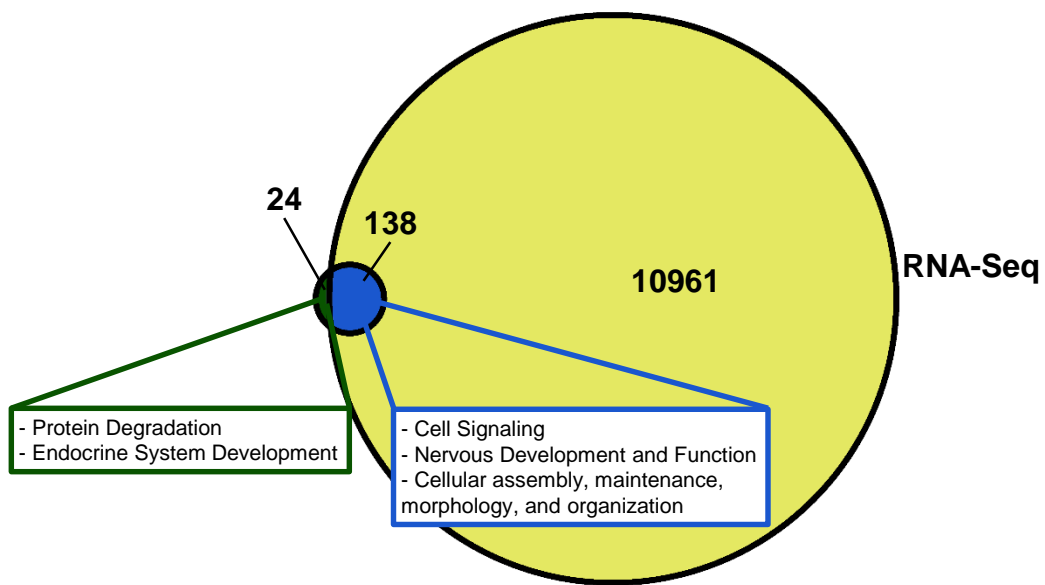

Supplement: Supplemental Figure 1 — Protein Ubiquitination Pathway. One of top four canonical pathways predicted by Ingenuity Pathway Analysis for H3K9me3 enriched genes in baboon SVZ cells. The shaded focus genes (red highlight) were enriched with H3K9me3 identified by ChIP-Seq analysis. The node shape reflects the role of each element in the pathway and the direction and arrowhead shapes of each edge represent different types of interactions (see key at bottom-left panel). [file Presentation1.PDF]
