# Supplementary material for: Molecular targets of chromatin repressive mark H3K9me3 in primate progenitor cells within adult neurogenic niches
Source: Front Genet. 2014 Jul 30;5:252. doi: 10.3389/fgene.2014.00252 (PMC4115620; doi:10.3389/fgene.2014.00252)
Supplement: Supplemental Table 1 — (i) Summary of Enriched Loci of H3K9me3; (ii) Gene Ontology by DAVID for H3K9me3-enriched genes in Baboon SVZ Cells; (iii) Integrated analyses for ChIP-Seq and RNA-Seq; (iv) A subset of H3K9me3-enriched genes was characterized as imprinting genes. [file Presentation2.PDF]

Supplemental Table 1 (i): Enriched Loci of H3K9me3

Gene Name (Rhesus)	RefSeq ID (Rhesus)	Gene Name (Rhesus)	RefSeq ID (Rhesus)	Gene Name (Rhesus)	RefSeq ID (Rhesus)	Gene Name (Rhesus)	RefSeq ID (Rhesus)
HMX1	NM_001172429	MOSC2	NM_001257477	SLC2A3	NM_001261533	EEF1B2	NM_001266385
CTBP1	NM_001266780	LYPLAL1	NM_001266865	TAS2R13	NM_001080767	GPR1	NM_001047129
PCGF3	NM_001258171	LOC100499547	NM_001195058	TAS2R46	NM_001080757	LANCL1	NM_001258121
ISG15	NM_001266806	TNNT2	NM_001247991	SLCO1A2	NM_001261711	IKZF2	NM_001258078
VWA1	NM_001260638	CAMSAP1L1	NM_001266713	FAR2	NM_001265709	KIAA1486	NM_001194144
SSU72	NM_001261547	ASPM	NM_001104535	METTL20	NM_001193718	PID1	NM_001265883
TPRG1L	NM_001260515	RGS2	NM_001265925	CNTN1	NM_001261751	PSMD1	NM_001257805
CTNNBIP1	NM_001260588	C1H1orf21	NM_001260863	ADAMTS20	NM_001110539	UGT1A1	NM_001032869
DFFA	NM_001260683	FAM20B	NM_001260759	TMEM117	NM_001261543	AGAP1	NM_001261633
DHRS3	NM_001260537	RGS5	NM_001260517	LMBR1L	NM_001194754	UBE2F	NM_001260974
EFHD2	NM_001265791	ASTN1	NM_001266732	TMBIM6	NM_001195449	PDCD1	NM_001114358
NECAP2	NM_001258172	PGBD2	NM_001258110	SLC4A8	NM_001258188	MYT1L	NM_001265731
SH2D5	NM_001266317	NLRP3	NM_001114351	MAP3K12	NM_001260812	RNASEH1	NM_001257858
ALPL	NM_001266869	SMYD3	NM_001265933	STAC3	NM_001194695	RPS7	NM_001266796
PITHD1	NM_001260794	COX20	NM_001257785	GEMIN7	NM_001194715	IAH1	NM_001193736
FOXJ3	NM_001266996	GREM2	NM_001266108	USP15	NM_001266957	ROCK2	NM_001260913
BEND5	NM_001266827	RHOU	NM_001265717	XPOT	NM_001258184	FAM49A	NM_001193919
JUN	NM_001265850	ADNP	NM_001266818	LLPH	NM_001266795	TTC32	NM_001257581
ATG4C	NM_001257887	SPATA2	NM_001260963	DYRK2	NM_001261216	C13H2orf44	NM_001194093
PDE4B	NM_001265964	CSE1L	NM_001257813	MDM2	NM_001266402	DNAJC27	NM_001257595
LRRC40	NM_001261023	MATN4	NM_001168628	LYZ	NM_001101733	KIF3C	NM_001266930
NEGR1	NM_001261637	GDAP1L1	NM_001257531	TPH2	NM_001039946	RASGRP3	NM_001261700
TNNI3K	NM_001204800	VSTM2L	NM_001260601	PPP1R12A	NM_001266093	CYP1B1	NM_001266868
PTGFR	NM_001266439	SNTA1	NM_001266239	PPFIA2	NM_001266465	SRSF7	NM_001195586
CDC7	NM_001266610	NSFL1C	NM_001266663	DDT	NM_001257668	SLC8A1	NM_001032861
TMED5	NM_001266841	C10H20orf196	NM_001260687	TMCC1	NM_001261150	MTA3	NM_001265625
CNN3	NM_001261809	PLCB1	NM_001257867	EPYC	NM_001194215	PPM1B	NM_001266202
RWDD3	NM_001266129	FLRT3	NM_001265872	CDK17	NM_001194763	GPR75	NM_001204509
AMY2B	NM_001266186	PET117	NM_001266528	TMPO	NM_001261527	RTN4	NM_001205127
PSRC1	NM_001266394	THBD	NM_001266049	SLC25A3	NM_001257587	PEX13	NM_001258170
RAP1A	NM_001261638	TMEM90B	NM_001194092	FAM71C	NM_001193809	XPO1	NM_001266844
SIKE1	NM_001261494	NANP	NM_001266157	SCYL2	NM_001266890	SERTAD2	NM_001261684
FAM46C	NM_001266603	CECR5	NM_001194230	MED13L	NM_001261532	RAB1A	NM_001257272
NOTCH2	NM_001260662	PEX26	NM_001260660	RFC5	NM_001261662	FAM136A	NM_001267012
TXNIP	NM_001257935	KLHL22	NM_001261588	PIWIL1	NM_001195711	MPHOSPH10	NM_001266435
LOC717324	NM_001257530	TOP3B	NM_001266407	NOC4L	NM_001258179	STAMBP	NM_001260604
SPRR1B	NM_001032963	ZNF280B	NM_001261022	PXMP2	NM_001261587	POLE4	NM_001261810
ILF2	NM_001266173	SNRPD3	NM_001194052	SPOPL	NM_001266382	CKB	NM_001267031
HAX1	NM_001266445	CRYBB2	NM_001122894	GTDC1	NM_001261488	LRRTM1	NM_001257467
THBS3	NM_001260514	RASL10A	NM_001260903	MMADHC	NM_001265642	ST3GAL5	NM_001257500
FCGR2A	NM_001257300	RBFOX2	NM_001261516	NMI	NM_001257642	RNF103	NM_001266577
FCGR2B	NM_001257302	ATF4	NM_001266632	ARL6IP6	NM_001260607	TEX37	NM_001193977
UAP1	NM_001266909	EP300	NM_001266486	RPRM	NM_001193741	IMPACT	NM_001266051
RNF187	NM_001164330	TSPO	NM_001258111	GORASP2	NM_001261707	KCNIP3	NM_001257864
C1H1orf35	NM_001265942	CERK	NM_001266852	PDE1A	NM_001257584	ACTR1B	NM_001257417
ARF1	NM_001193287	ZBED4	NM_001261128	COL3A1	NM_001266039	INPP4A	NM_001261619
ADCK3	NM_001258190	RABL2B	NM_001260670	NAB1	NM_001257641	UNC50	NM_001195448
TIMM8B	NM_001267792	VWF	NM_001243086	SDPR	NM_001265645	LYG1	NM_001194261
TLR5	NM_001130429	PIANP	NM_001194405	SLC39A10	NM_001260918	IL1R2	NM_001047134
EMP2	NM_001265948	CACNG3	NM_001261544	ORC6L	NM_001194605	CDH8	NM_001257905
ZC3H7A	NM_001257840	LCMT1	NM_001260979	PHKB	NM_001261044	CBFB	NM_001266558
GDE1	NM_001260575	TUFM	NM_001266084	MT3	NM_001266138	NUDT7	NM_001194315
CDR2	NM_001260929	C20H16orf54	NM_001194257	ARL2BP	NM_001267028	SDR42E1	NM_001194527
UBFD1	NM_001266120	C20H16orf58	NM_001194596	GINS3	NM_001260655	MPHOSPH6	NM_001193603
TMEM30A	NM_001260817	MCHR2	NM_001032948	ASF1A	NM_001194486	PSMB1	NM_001260636
MYO6	NM_001104536	C4H6orf203	NM_001266984	SLC35F1	NM_001266534	GPR78	NM_001193900
ELOVL4	NM_001040419	GINM1	NM_001193728	NT5DC1	NM_001261622	LYAR	NM_001258141
TPBG	NM_001193701	UST	NM_001257513	HS3ST5	NM_001193687	ZNF518B	NM_001261812
MRAP2	NM_001261086	FBXO30	NM_001261073	C4H6orf225	NM_001193364	NKX3-2	NM_001194111
POLR1D	NM_001257459	VTA1	NM_001261520	FIG4	NM_001172433	LCORL	NM_001172360
PNRC1	NM_001266366	IFNGR1	NM_001266300	PLG	NM_001043075	ANAPC4	NM_001266920
GJA10	NM_001193951	MED23	NM_001261479	PACRG	NM_001266357	RBPJ	NM_001260693
FUT9	NM_001194065	CENPW	NM_001193377	RNASET2	NM_001266725	TBC1D19	NM_001260783
FBXL4	NM_001266033	GJA1	NM_001261067	CCR6	NM_001032935	C5H4orf19	NM_001257674

Supplemental Table 1 (i): Enriched Loci of H3K9me3 (Continued)

Gene Name (Rhesus)	RefSeq ID (Rhesus)	Gene Name (Rhesus)	RefSeq ID (Rhesus)	Gene Name (Rhesus)	RefSeq ID (Rhesus)	Gene Name (Rhesus)	RefSeq ID (Rhesus)
C13H2orf40	NM_001194457	LCN9	NM_001171841	TOM1L1	NM_001261224	NOL4	NM_001194221
TTL	NM_001266278	SURF6	NM_001194177	VMP1	NM_001190897	RIT2	NM_001257707
HNMT	NM_001195823	UCK1	NM_001194319	PPM1D	NM_001260836	ST8SIA5	NM_001083950
TSN	NM_001266679	URM1	NM_001266132	HNF1B	NM_001194548	CXXC1	NM_001266065
POLR2D	NM_001267787	OR1Q1	NM_001193814	C16H17orf96	NM_001193705	SMAD4	NM_001261090
MZT2B	NM_001193944	DBC1	NM_001260978	KRTAP4-1	NM_001193889	TXNL1	NM_001261708
IMP4	NM_001266231	POLE3	NM_001257857	TMEM106A	NM_001194813	PMAIP1	NM_001260640
CFC1	NM_001190851	SLC31A1	NM_001257506	KCNJ2	NM_001032859	CDH7	NM_001193994
FAM168B	NM_001194008	TXN	NM_001042732	RAB37	NM_001266608	CD226	NM_001042643
CHID1	NM_001266971	OR13C8	NM_001194361	PRPSAP1	NM_001260586	CNDP2	NM_001257758
TOLLIP	NM_001261271	PPP3R2	NM_001195420	NPTX1	NM_001266044	PQLC1	NM_001265722
CARS	NM_001266326	TMOD1	NM_001265684	FN3KRP	NM_001194815	TXNL4A	NM_001260866
PGA4	NM_001104594	ACO1	NM_001257865	MRP63	NM_001193743	C2CD4C	NM_001194820
OR5AN1	NM_001193824	CAAP1	NM_001194328	SAP18	NM_001261034	GNG7	NM_001193395
OR10Q1	NM_001193880	MTAP	NM_001261737	PSPC1	NM_001257902	VMAC	NM_001193782
TIMM10	NM_001261523	IFNA13	NM_001194367	MTMR6	NM_001267015	MCOLN1	NM_001260540
OR4B1	NM_001194287	SNAPC3	NM_001260804	SLC7A1	NM_001266615	IER2	NM_001266014
SLC35C1	NM_001258150	RANBP6	NM_001194574	STARD13	NM_001261243	CYP4F22	NM_001194539
API5	NM_001257933	JAK2	NM_001265901	C13orf36	NM_001193731	CYP4F12	NM_001194560
FBXO3	NM_001257701	SLC1A1	NM_001265831	HTR2A	NM_001032966	LPAR2	NM_001266718
ARL14EP	NM_001261159	CBWD1	NM_001193953	ALG11	NM_001190890	PLEKHF1	NM_001193737
METTL15	NM_001260955	KLF9	NM_001265918	OR5L1	NM_001194579	LSM14A	NM_001257954
SVIP	NM_001261697	CYP1D1	NM_001246671	PCDH17	NM_001265711	SCN1B	NM_001258167
RPS13	NM_001265917	NTRK2	NM_001261297	PCDH9	NM_001258186	HCST	NM_001032835
C14H11orf58	NM_001193864	HSPA5	NM_001266596	KLHL1	NM_001195300	COX7AH	NM_001040278
RNF141	NM_001266485	CENPP	NM_001194801	TBC1D4	NM_001172423	ZNF790	NM_001261671
SWAP70	NM_001172414	OGN	NM_001261240	FBXL3	NM_001261635	ZNF345	NM_001194827
RIC3	NM_001261177	HIATL1	NM_001194284	SLITRK1	NM_001260787	SUPT5H	NM_001266951
OR10A6	NM_001194102	HSD17B3	NM_001266504	SOX21	NM_001193732	AKT2	NM_001265989
OR2D3	NM_001194381	GLOD4	NM_001265952	DNAJC3	NM_001266404	RABAC1	NM_001260923
OR52W1	NM_001194213	BHLHA9	NM_001194728	STK24	NM_001266859	PRR19	NM_001194317
TRIM22	NM_001113359	INPP5K	NM_001260646	TEX30	NM_001266631	IRGQ	NM_001265865
HBB	NM_001164428	NLRP1	NM_001114349	ARGLU1	NM_001194022	C19orf61	NM_001194356
FOLR1	NM_001194647	WSCD1	NM_001257765	F7	NM_001080136	PVR	NM_001043386
FCHSD2	NM_001265957	PLSCR3	NM_001265888	F10	NM_001104584	FOXA3	NM_001194519
RAB6A	NM_001261303	CNTROB	NM_001261206	CDC16	NM_001266466	NAPA	NM_001194588
RPS3	NM_001266290	RNF222	NM_001194585	ZNF828	NM_001260800	BAX	NM_001261016
RPS2	NM_001193577	STX8	NM_001265754	MC5R	NM_001193897	PPP2R1A	NM_001257922
FAM181B	NM_001194034	TVP23B	NM_001260706	PSMG2	NM_001193620	TFPT	NM_001194720
CCDC90B	NM_001260811	ZNF287	NM_001266709	NAPG	NM_001258101	LILRA3	NM_001040675
TMEM126B	NM_001267027	SHMT1	NM_001257431	TWSG1	NM_001257525	KIR3DL12	NM_001105171
CTSC	NM_001195651	MAP2K3	NM_001265947	NDUFV2	NM_001257585	KIRDL8	NM_001105172
CHORDC1	NM_001257756	WSB1	NM_001261284	ZFP161	NM_001265870	LOC100125569	NM_001105176
C14H11orf75	NM_001193371	LYRM9	NM_001193351	NDC80	NM_001265902	FCAR	NM_001039950
BIRC2	NM_001261321	GOSR1	NM_001257945	YES1	NM_001257512	NLRP8	NM_001114115
PTP4A2	NM_001266125	CCL4L1	NM_001032873	RBBP8	NM_001258203	ZNF671	NM_001260628
VPS26B	NM_001257745	CCL11	NM_001032874	SS18	NM_001257494	UBE2M	NM_001194143
ZMYND19	NM_001194731	MRPL10	NM_001260951	CHST9	NM_001261472	GBE1	NM_001266287
C9orf167	NM_001193807	IGF2BP1	NM_001193921	TTR	NM_001261679	TOMM70A	NM_001266153
HSBP1	NM_001258044	KRTAP12-4	NM_001194238	HSPA13	NM_001193708	ACTB	NM_001033084
FOXC2	NM_001198708	PKNOX1	NM_001261289	ABCC13	NM_001032930	BRI3	NM_001266634
DPEP1	NM_001260742	ATP5J	NM_001257938	FAM20C	NM_001193713	ACHE	NM_001128088
PRMT2	NM_001265880	C3H21orf91	NM_001194344	COX19	NM_001257834	RABL5	NM_001194410
YBEY	NM_001265798	BTG3	NM_001266041	PAPOLB	NM_001194858	GATSL2	NM_001266104
KLF3	NM_001261280	ALB	NM_001195649	NFKB1	NM_001266053	TMEM184C	NM_001260921
TMEM156	NM_001193846	SULT1B1	NM_001193512	TACR3	NM_001037862	ARFIP1	NM_001266175
RHOH	NM_001260809	STAP1	NM_001194445	HADH	NM_001261209	LRAT	NM_001193939
ATP8A1	NM_001265879	LPHN3	NM_001261704	ENPEP	NM_001266656	PPID	NM_001265834
GRXCR1	NM_001194126	LOC100499503	NM_001194967	TRAM1L1	NM_001261483	NPY1R	NM_001032866
GNPDA2	NM_001194875	USP46	NM_001266963	MAD2L1	NM_001195813	SC4MOL	NM_001260803
OCIAD2	NM_001266991	MAPK10	NM_001260857	ANKRD50	NM_001266441	FBXO8	NM_001265874
PLAC8	NM_001258163	HPGDS	NM_001194212	SLC25A31	NM_001194300	AGA	NM_001199093
STBD1	NM_001194876	TSPAN5	NM_001194290	BANF1	NM_001193373	ODZ3	NM_001193929
CXCL9	NM_001032936	DDIT4L	NM_001194403	ABCE1	NM_001266954	IRF2	NM_001135793

Supplemental Table 1 (i): Enriched Loci of H3K9me3 (Continued)

Gene Name (Rhesus)	RefSeq ID (Rhesus)	Gene Name (Rhesus)	RefSeq ID (Rhesus)	Gene Name (Rhesus)	RefSeq ID (Rhesus)	Gene Name (Rhesus)	RefSeq ID (Rhesus)
ALCAM	NM_001257702	PSMA2	NM_001266881	HTR1A	NM_001198700	MEF2A	NM_001260914
CD47	NM_001266517	RALA	NM_001266456	MAP1B	NM_001265687	WDR12	NM_001260900
DPPA2	NM_001266584	NPSR1	NM_001032942	ENC1	NM_001260583	SCFD1	NM_001266523
B4GALT4	NM_001257832	SP4	NM_001266828	AGGF1	NM_001261037	HECTD1	NM_001261259
CSTA	NM_001258142	FERD3L	NM_001194245	LHFPL2	NM_001194391	ARHGAP5	NM_001198663
OXTR	NM_001044732	TMEM106B	NM_001193700	RPS23	NM_001193580	SEC23A	NM_001261363
CAV3	NM_001194078	ICA1	NM_001265706	CETN3	NM_001266092	CTAGE5	NM_001195820
FAM19A1	NM_001261362	COL1A2	NM_001266337	ARRDC3	NM_001260940	RPL10L	NM_001194068
SUCLG2	NM_001261121	BET1	NM_001260728	RIOK2	NM_001260597	RPS29	NM_001267537
SLC25A26	NM_001261056	SEMA3C	NM_001260533	RGMB	NM_001265620	FERMT2	NM_001266319
C2H3orf14	NM_001257952	IMMP2L	NM_001261314	FAM174A	NM_001194071	PSMA3	NM_001260896
FAM107A	NM_001257546	GPR85	NM_001266820	ST8SIA4	NM_001266232	JKAMP	NM_001260993
RBM15B	NM_001265669	TAS2R16	NM_001080759	GIN1	NM_001194084	TRMT5	NM_001261618
ZNF501	NM_001194298	GPR37	NM_001257869	EFNA5	NM_001265728	RHOJ	NM_001260613
SNRK	NM_001257661	GCC1	NM_001265763	STARD4	NM_001260934	GPHN	NM_001257815
LYZL4	NM_001194683	MTPN	NM_001260744	DCP2	NM_001266464	FAM71D	NM_001194896
MOBP	NM_001190966	C3H7orf55	NM_001194423	TSSK1B	NM_001193468	RGS6	NM_001265721
RPSA	NM_001195480	RAB19	NM_001261554	CDO1	NM_001260608	PNMA1	NM_001257567
DNAJC19	NM_001266267	C3H7orf33	NM_001194007	DTWD2	NM_001261379	AHSA1	NM_001266884
ACTL6A	NM_001104559	EZH2	NM_001266503	CSNK1G3	NM_001258050	FLRT2	NM_001266473
KCNMB2	NM_001194444	GIMAP4	NM_001257713	GRAMD3	NM_001265990	GALC	NM_001044262
NLGN1	NM_001257734	ACTR3B	NM_001266189	LRRTM2	NM_001266604	TDP1	NM_001257779
SEC62	NM_001195502	NCAPG2	NM_001258148	RNF14	NM_001260813	CPSF2	NM_001257580
PDCD10	NM_001266410	DUSP22	NM_001260542	NDFIP1	NM_001194017	GOLGA5	NM_001257502
SPTSSB	NM_001193354	EXOC2	NM_001266944	CSNK1A1	NM_001261235	DICER1	NM_001257872
MLF1	NM_001261200	GMDS	NM_001266789	CCDC69	NM_001260862	GLRX5	NM_001265635
GMPS	NM_001261203	TMEM170B	NM_001261045	GLRA1	NM_001194497	GSKIP	NM_001194191
FAIM	NM_001266110	RNF144B	NM_001265699	C6H5orf4	NM_001257519	CINP	NM_001266052
SOX14	NM_001194657	GMNN	NM_001258199	CNOT8	NM_001257959	CRIP1	NM_001266846
TMEM108	NM_001265634	ZNF322A	NM_001265932	GABRG2	NM_001261778	ZNF596	NM_001266814
RAB5A	NM_001261148	HIST1H2BK	NM_001260530	MAT2B	NM_001257473	CLN8	NM_001194050
UBE2E2	NM_001193865	HIST1H2AK	NM_001194031	RARS	NM_001261218	AGPAT5	NM_001257833
CMC1	NM_001261273	OR2J3	NM_001194199	ATP6V0E1	NM_001261126	MNP1A	NM_001032862
AZI2	NM_001266975	OR5V1	NM_001194206	DRD1	NM_001206975	SPAG11B	NM_001037282
TGFBR2	NM_001261151	MAMU-F	NM_001042770	THOC3	NM_001257489	LOC574310	NM_001032922
CMTM7	NM_001260694	C4H6orf136	NM_001105163	B4GALT7	NM_001260676	MNP2	NM_001032863
CCR4	NM_001266020	POU5F1	NM_001114955	MGAT1	NM_001257830	ROAD2	NM_001131060
EIF4A2	NM_001195661	MAMU-I	NM_001079671	NIPA2	NM_001194886	CTSB	NM_001194899
CLDN1	NM_001193969	MAMU-B	NM_001048245	NDN	NM_001172102	FDFT1	NM_001260724
PYDC2	NM_001193432	MICB	NM_001114956	SNRPN	NM_001261205	TNKS	NM_001261281
CEP19	NM_001260983	CD9	NM_001114965	UBE3A	NM_001261040	TUSC3	NM_001266279
LOC711340	NM_001265954	MAMU-DRB1	NM_001044261	GABRB3	NM_001260702	FGF17	NM_001194186
TFRC	NM_001257303	MAMU-DMB	NM_001265654	NDNL2	NM_001257507	NEFL	NM_001265845
ITFG3	NM_001266354	RPL10A	NM_001266226	EMC4	NM_001260989	LEPROTL1	NM_001266983
DECR2	NM_001204462	TREML1	NM_001194729	ZNF770	NM_001261541	MAK16	NM_001260675
C20H16orf13	NM_001204163	CCND3	NM_001265987	ATPBD4	NM_001266595	ERLIN2	NM_001257541
GNPTG	NM_001204748	CDC5L	NM_001261272	CTDSPL2	NM_001266809	IDO1	NM_001077483
FAM86A	NM_001194839	SUPT3H	NM_001266569	DTWD1	NM_001260730	THAP1	NM_001257879
TMEM114	NM_001194176	RHAG	NM_001032815	RSL24D1	NM_001258062	MCM4	NM_001266203
WBSCR17	NM_001265889	IL17F	NM_001261287	RAB27A	NM_001193855	UBE2V2	NM_001266843
VKORC1L1	NM_001266931	UBE2E3	NM_001257571	HEXA	NM_001261529	SNAI2	NM_001260982
CHCHD2	NM_001198739	LMBRD1	NM_001194516	PML	NM_001042434	LOC100499551	NM_001195073
MRPS17	NM_001266196	C4H6orf57	NM_001194396	SCAMP5	NM_001260562	GGH	NM_001260712
GRB10	NM_001257428	KCNQ5	NM_001265828	SIN3A	NM_001266100	ARFGEF1	NM_001261046
CYP4V2	NM_001193838	BRIX1	NM_001261247	FBXO22	NM_001266238	PI15	NM_001193804
FRG1	NM_001261228	SLC1A3	NM_001266066	STARD5	NM_001260916	STMN2	NM_001265775
EXOC3	NM_001260656	PTGER4	NM_001266046	C7H15orf40	NM_001261261	MRPS28	NM_001261089
CCDC127	NM_001194083	C6H5orf51	NM_001193823	RPS17L	NM_001265804	CHMP4C	NM_001193985
CCT5	NM_001258189	GHR	NM_001042667	PDE8A	NM_001266644	RALYL	NM_001201567
FAM105A	NM_001193791	SEPP1	NM_001159490	SEC11A	NM_001261576	CA2	NM_001195417
FAM105B	NM_001193800	FGF10	NM_001193950	NRGN	NM_001266787	DCAF4L2	NM_001198702
FAM134B	NM_001261775	PELO	NM_001190867	SV2B	NM_001260629	NBN	NM_001265739
LOC100499546	NM_001195043	IL6ST	NM_001265991	RGMA	NM_001266917	LRRC69	NM_001193750
DROSHA	NM_001261157	DEPDC1B	NM_001194881	IGF1R	NM_001261352	MTDH	NM_001266369

Supplemental Table 1 (i): Enriched Loci of H3K9me3 (Continued)

Gene Name (Rhesus)	RefSeq ID (Rhesus)	Gene Name (Rhesus)	RefSeq ID (Rhesus)	Gene Name (Rhesus)	RefSeq ID (Rhesus)	Gene Name (Rhesus)	RefSeq ID (Rhesus)
LRP12	NM_001261683	TMSB4X	NM_001260557	GOLGA7B	NM_001260720	MRPS16	NM_001257904
SYBU	NM_001265668	GEMIN8	NM_001257640	CWF19L1	NM_001266734	TSPAN15	NM_001266979
UTP23	NM_001193908	EIF1AX	NM_001261777	GSTO1	NM_001260555	DDX50	NM_001258182
TRIB1	NM_001193695	DCAF8L2	NM_001266964	XPNPEP1	NM_001266962	CCAR1	NM_001260782
ST3GAL1	NM_001261634	MAGEB1	NM_001260599	GPAM	NM_001266771	LRRTM3	NM_001266575
PTP4A3	NM_001193694	FTHL17	NM_001194051	HTRA1	NM_001258176	CISD1	NM_001257590
ZNF250	NM_001267795	TMEM47	NM_001266555	GPR26	NM_001265705	MBL2	NM_001105535
ZMYND11	NM_001261702	PRRG1	NM_001261082	OAT	NM_001266478	CSTF2T	NM_001266798
LARP4B	NM_001260689	SERPINB6	NM_001202551	C9H10orf122	NM_001190891	PTEN	NM_001260965
WDR37	NM_001260851	MAGED4B	NM_001266405	BCCIP	NM_001265671	CYP2C75	NM_001040211
AKR1E2	NM_001194235	HUWE1	NM_001266752	NKX6-2	NM_001266630	PLS3	NM_001266221
KIN	NM_001261813	SPIN3	NM_001260981	CYP2E1	NM_001040213	C1GALT1C1	NM_001260840
DCLRE1C	NM_001204350	ZC4H2	NM_001261736	APOBEC3A	NM_001246231	THOC2	NM_001260621
CACNB2	NM_001257782	EDA2R	NM_001193467	MAMU-AG	NM_001134230	CXHXorf64	NM_001193842
ARL5B	NM_001193479	AR	NM_001032911	MRPL41	NM_001258134	FAM127A	NM_001194127
DNAJC1	NM_001261677	CXCR3	NM_001145040	MAMU-B18	NM_001114964	DDX26B	NM_001265682
RAB18	NM_001266790	RLIM	NM_001266839	COX7A2L	NM_001266821	MAGEC2	NM_001265825
ARHGAP12	NM_001260799	CXHXorf26	NM_001194106	ZBED1	NM_001266708	SPANXN	NM_001032954
LOC100499557	NM_001195085	FGF16	NM_001194115	NLGN4Y	NM_001145058	SLITRK2	NM_001261220
CSGALNACT2	NM_001172170	PGK1	NM_001195658	PNPLA4	NM_001193844	CD99L2	NM_001261370
ZNF32	NM_001260922	P2RY10	NM_001258158	AMELX	NM_001098513	NXT2	NM_001265707
ZNF22	NM_001261786	POU3F4	NM_001194259	ANXA7	NM_001260935	H2BFWT	NM_001193918
OGDHL	NM_001265802	TGIF2LX	NM_001032888	FAM190B	NM_001193762	NAP1L3	NM_001265658
SNCG	NM_001266560	PABPC5	NM_001044728	GRID1	NM_001257667		

Supplemental Table 1 (ii): Enriched Loci of H3K9me3 Gene Ontology

Term	Count	%	PValue	Expected	Genes	Fold Enrichment	Bonferroni	Benjamini	FDR
Acetylation	162	1.8135005	4.41E-08	1.07E+02	XPO1, MEF2A, CMC1, CNDP2, CBFB, RAB1A, NT5DC1, ZNF828, SIN3A, CISD1, MAK16, HIST1H2BK, HADH, SUPT5H, FBXO22, API5, GNG7, CARS, SUCLG2, DFFA, SNRPN, BEND5, CECR5, TIMM8B, CTDSPL2, TNNT2, PSMA2, EP300, SLC25A31, MAD2L1, CCR6, RAB18, HUWE1, RARS, PSMA3, RPS13, CHORDC1, EEF1B2, HAX1, ACTR3B, PABPC5, UBE2V2, RIC3, ARFGEF1, CXXC1, POLE4, RPS29, PSMB1, AGGF1, CSE1L, POLE3, MTPN, BRIX1, RPL10A, TMEM30A, RPS23, XPOT, RPSA, LCMT1, CRIP1, UAP1, MRPS28, IKZF2, MMADHC, MAP2K3, MPHOSPH10, SMAD4, SAP18, TSN, GLOD4, PRPSAP1, LARP4B, OCIAD2, RPS7, CCT5, GBE1, TOMM70A, TMPO, AHSA1, KLF3, RTN4, TOLLIP, PHKB, NFKB1, RPS2, PTEN, RPS3, FAM168B, LSM14A, EFHD2, SDPR, RANBP6, ACTR1B, TRMT5, PSMD1, KLHL22, SLC25A3, RAB6A, PLCB1, USP15, DNAJC19, MT3, ATP5J, CRYBB2, PPP2R1A, ABCE1, ZNF280B, POLR1D, DECR2, ADNP, UBE2F, CDC5L, UGT1A1, MCM4, GMPS, ATG4C, EIF4A2, UBE2M, DDT, TXN, RAB5A, MTAP, TNNI3K, TMSB4X, CA2, NRGN, THOC3, TUFM, LANCL1, FAM136A, COX7A2L, POLR2D, ANXA7, SNRK, TBC1D4, WDR12, EXOC3, ACTL6A, HSPA5, LYPLAL1, GSTO1, HBB, NEFL, EXOC2, ACTB, CSNK1A1, SHMT1, GIMAP4, PDCD10, GMDS, VTA1, GMNN, MAP1B, XPNPEP1, PLEKHF1, SCFD1, RAB37, BAX, PSPC1, IRF2, HIST1H2AK, PGK1	1.508379158	2.38E-05	2.38E-05	6.42E-05
Ras GTPase	18	0.20150006	7.77E-05	5.88E+00	RHOJ, RHOU, RAB1A, RABL2B, DNAJC27, ARHGAP5, RAB37, RAB18, RAB19, RASL10A, RAB5A, RALA, RAP1A, RIT2, RAB6A, AGAP1, RAB27A, RHOH	3.063724138	0.090272099	0.090272099	0.12582077
Lipoprotein	47	0.52613904	1.55E-04	2.62E+01	RHOJ, ALPL, DRD1, ACHE, TACR3, PHKB, PPP3R2, LPAR2, RHOU, RAB1A, KCNIP3, RGMA, CD9, RGMB, HTR1A, RNF103, FOLR1, GORASP2, RASL10A, MC5R, RALA, RAB6A, YES1, ARL5B, NEGR1, DPEP1, GNG7, RAB27A, RHOH, GABRG2, NPY1R, CCDC69, PLG, TFRC, RAB18, PTP4A3, ARF1, RAB37, GOLGA7B, RAB19, LRP12, PLSCR3, PTP4A2, RAB5A, CNTN1, RAP1A, EFNA5	1.796134926	0.080370399	0.041026798	0.22523453
Protein transport and targeting	44	0.49255569	1.62E-04	2.40E+01	GNPTG, CAV3, MBL2, HS3ST5, FUT9, UNC50, BET1, RIC3, RHOU, RAB1A, KCNIP3, RABAC1, ST3GAL1, ST3GAL5, GORASP2, TNKS, RAB6A, GOLGA5, B4GALT7, HPGDS, B4GALT4, SCAMP5, SEC23A, ICA1, MYO6, WBSCR17, UST, NSFL1C, NDFIP1, GCC1, FAM134B, MGAT1, SLC35C1, SCFD1, ARF1, TOM1L1, GOLGA7B, SCYL2, ST8SIA4, CHST9, CSGALNACT2, ST8SIA5, SVIP, GOSR1	1.835910385	0.083896467	0.028786182	0.23554959
Axon	18	0.20150006	5.90E-04	6.97E+00	ACTB, SNCG, DRD1, AR, MYO6, GABRB3, STMN2, MAP1B, NPY1R, ALCAM, HTR1A, MTPN, LRRTM1, NTRK2, RAB5A, CA2, NEFL, MAP3K12	2.583962264	0.232503016	0.123930948	0.8339533
Small GTP-binding protein	18	0.20150006	9.82E-04	7.27E+00	RHOJ, RHOU, RAB1A, RABL2B, DNAJC27, ARF1, RAB37, RAB18, RAB19, RAB5A, RALA, RAP1A, RIT2, RAB6A, AGAP1, ARL5B, RAB27A, RHOH	2.476663225	0.697321976	0.328580995	1.57775696
Cell soma	18	0.20150006	0.00110078	7.36E+00	RTN4, SNCG, DRD1, MYO6, NDN, CNN3, MAP1B, KCNJ2, RAB1A, KLHL1, ALCAM, SLC1A3, HTR1A, CCR4, NTRK2, RAB5A, NEGR1, GHR	2.445535714	0.389466558	0.116050684	1.54941358
Small ribosomal subunit	10	0.11194448	0.00157511	2.76E+00	RPSA, MRPS17, MRPS16, MRPS28, RPS29, RPS13, RPS2, RPS23, RPS3, RPS7	3.623015873	0.506486393	0.131719943	2.21014986
Short sequence motif:Effector region	12	0.13433337	0.00173055	3.90E+00	RHOJ, RAB18, RAB37, RAB19, RASL10A, RAB5A, RALA, RAP1A, RAB6A, RAB1A, RAB27A, RHOH	3.07943072	0.970488896	0.970488896	2.93973413
Prenylation	16	0.17911116	0.00178992	6.36E+00	RHOJ, PHKB, RAB1A, RAB37, PTP4A3, RAB18, RAB19, RASL10A, PTP4A2, RAB5A, RALA, RAP1A, RAB6A, RAB27A, GNG7, RHOH	2.516352695	0.620618449	0.149163653	2.57471526
Methylation	21	0.2350834	0.00221626	9.86E+00	RHOJ, ACTB, SNRPD3, EP300, HIST1H2BK, RAB37, RAB18, RAB19, GORASP2, RASL10A, PIWIL1, RAP1A, RALA, HIST1H2AK, RAB6A, SUPT5H, GOLGA5, RAB27A, GNG7, RHOH, CRYBB2	2.129021547	0.698904143	0.157578865	3.17881607
Neuron projection	28	0.31344453	0.00223119	1.50E+01	SNCG, DRD1, CNN3, GABRB3, KCNJ2, KLHL1, ALCAM, IGF1R, HTR1A, SLC1A3, INPP5K, MTPN, LRRTM1, NEGR1, NEFL, IFNGR1, RAB27A, ACTB, AR, MYO6, STMN2, MAP1B, NPY1R, NTRK2, RAB5A, CA2, MAP3K12, HTR2A	1.86871345	0.632374919	0.153615849	3.11728619
Lipid moiety-binding region:S-geranylgeranyl cysteine	12	0.13433337	0.0030524	4.18E+00	RAB18, RAB37, RAB19, RASL10A, RAB5A, RALA, RAP1A, RAB6A, RAB1A, RAB27A, GNG7, RHOH	2.868097239	0.998006918	0.955356056	5.13015021
Ribosome biogenesis	8	0.08955558	0.0030976	1.96E+00	UTP23, FRG1, BRIX1, MPHOSPH10, WDR12, RSL24D1, ALG11, IMP4	4.089073129	0.813327947	0.189253816	4.41657963
Golgi membrane	18	0.20150006	0.00327762	8.15E+00	SEC23A, GNPTG, ICA1, UNC50, BET1, GJA1, ARFIP1, CYP2E1, ST3GAL1, MGAT1, ARF1, ST3GAL5, ST8SIA4, ST8SIA5, CSGALNACT2, GOSR1, GOLGA5, SCAMP5	2.208870968	0.770254724	0.189506544	4.54795175

Supplemental Table 1 (iii): H3K9me3 ChIP-Seq & RNA-Seq Overlap

H3K9me3 targets not detectable by RNA-Seq			
ABCC13	CYP4F22	LOC100125569	PML
ADAMTS20	DCAF4L2	LOC100499503	PPP3R2
AGA	DCAF8L2	LOC100499546	PQLC1
AKR1E2	DDIT4L	LOC100499547	PRR19
ALB	DDX26B	LOC100499551	PSRC1
ALG11	DEPDC1B	LOC100499557	PTGER4
ALPL	DPEP1	LOC574310	PTGFR
AMELX	DPPA2	LOC711340	PTP4A3
AMY2B	EDA2R	LOC717324	PYDC2
APOBEC3A	EFHD2	LPAR2	RAB19
AR	ENPEP	LRRC69	RAB27A
ARL6IP6	EPYC	LYG1	RAB37
ASPM	EZH2	LYZL4	RASL10A
BAX	F10	MAD2L1	RBBP8
BHLHA9	F7	MAGEB1	RBM15B
C10H20orf196	FAM105A	MAGEC2	RGMB
C13H2orf40	FAM105B	MAGED4B	RHAG
C13H2orf44	FAM181B	MAMU-AG	RHOH
C13orf36	FAM190B	MAMU-B	RHOJ
C14H11orf58	FAM19A1	MAMU-B18	RNASET2
C14H11orf75	FAM46C	MAMU-DMB	RNF144B
C16H17orf96	FAM71C	MAMU-DRB1	RNF222
C19orf61	FAM71D	MAMU-F	ROAD2
C1H1orf21	FAM86A	MAMU-I	RPL10L
C1H1orf35	FBXO22	MAP2K3	RPRM
C20H16orf13	FBXO8	MATN4	RPS17L
C20H16orf54	FCGR2A	MBL2	SC4MOL
C20H16orf58	FCGR2B	MC5R	SDPR
C2CD4C	FERD3L	MCHR2	SEMA3C
C2H3orf14	FGF16	MCOLN1	SLC25A31
C3H21orf91	FGF17	METTL15	SLC35C1
C3H7orf33	FOLR1	METTL20	SLCO1A2
C3H7orf55	FOXA3	MICB	SNAI2
C4H6orf136	FOXC2	MNP1A	SOX14
C4H6orf203	FTHL17	MNP2	SOX21
C4H6orf225	GATSL2	MOSC2	SPAG11B
C4H6orf57	GBE1	MRAP2	SPANXN
C5H4orf19	GIMAP4	MTPN	SPRR1B
C6H5orf4	GIN1	MZT2B	SPTSSB
C6H5orf51	GINS3	NDC80	ST8SIA5
C7H15orf40	GJA10	NKX3-2	STAC3
C9H10orf122	GLRA1	NLGN4Y	STAP1
C9orf167	GOLGA7B	NLRP1	STARD5
CAMSAP1L1	GPR26	NLRP3	STBD1
CAV3	GPR78	NLRP8	SULT1B1
CBFB	GREM2	NMI	SURF6
CBWD1	GRXCR1	NPSR1	TACR3
CCDC69	H2BFWT	NXT2	TAS2R13
CCL11	HBB	ODZ3	TAS2R16
CCL4L1	HCST	OGN	TAS2R46
CCR4	HIST1H2AK	OR10A6	TEX30
CCR6	HMX1	OR10Q1	TEX37
CD226	HNF1B	OR13C8	TGFBR2
CENPP	HPGDS	OR1Q1	TGIF2LX
CENPW	HSD17B3	OR2D3	THBD
CFC1	HTR1A	OR2J3	THOC3
CHID1	IDO1	OR4B1	TLR5
CHST9	IER2	OR52W1	TMEM108
CLDN1	IFNA13	OR5AN1	TMEM114
CMTM7	IGF2BP1	OR5L1	TMEM90B
COL1A2	IL17F	OR5V1	TNNI3K
COL3A1	IL1R2	ORC6L	TNNT2
COX20	ISG15	OXTR	TOP3B
COX7AH	ITFG3	P2RY10	TPH2
CRYBB2	KCNIP3	PABPC5	TREML1
CSTA	KCNMB2	PAPOLB	TSPO
CXCL9	KIAA1486	PDCD1	TSSK1B
CXCR3	KIR3DL12	PGA4	TTC32
CXHXorf26	KIRDL8	PGBD2	TTR
CXHXorf64	KLHL1	PI15	TVP23B
CYP1B1	KRTAP12-4	PIWIL1	UGT1A1
CYP1D1	KRTAP4-1	PLEKHF1	ZBED1
CYP2C75	LCN9	PLG	ZFP161
CYP2E1	LILRA3	PLSCR3	ZNF280B
CYP4F12	LMBR1L	PMAIP1	ZNF322A
ZNF828			

H3K9me3 targets detectable by RNA-Seq						
ABCE1	CINP	FERMT2	KIF3C	NRGN	RHOU	SUPT5H
ACHE	CISD1	FGF10	KIN	NSFL1C	RIC3	SV2B
ACO1	CKB	FIG4	KLF3	NT5DC1	RIOK2	SVIP
ACTB	CLN8	FLRT2	KLF9	NTRK2	RIT2	SWAP70
ACTL6A	CMC1	FLRT3	KLHL22	NUDT7	RLIM	SYBU
ACTR1B	CNDP2	FN3KRP	LANCL1	OAT	RNASEH1	TBC1D19
ACTR3B	CNN3	FOXJ3	LARP4B	OCIAD2	RNF103	TBC1D4
ADCK3	CNOT8	FRG1	LCMT1	OGDHL	RNF14	TDP1
ADNP	CNTN1	FUT9	LCORL	PACRG	RNF141	TFPT
AGAP1	CNTROB	GABRB3	LEPROTL1	PCDH17	RNF187	TFRC
AGGF1	COX19	GABRG2	LHFPL2	PCDH9	ROCK2	THAP1
AGPAT5	COX7A2L	GALC	LLPH	PCGF3	RPL10A	THBS3
AHSA1	CPSF2	GCC1	LMBRD1	PDCD10	RPS13	THOC2
AKT2	CRIP1	GDAP1L1	LPHN3	PDE1A	RPS2	TIMM10
ALCAM	CSE1L	GDE1	LRAT	PDE4B	RPS23	TIMM8B
ANAPC4	CSGALNACT2	GEMIN7	LRP12	PDE8A	RPS29	TMBIM6
ANKRD50	CSNK1A1	GEMIN8	LRRC40	PELO	RPS3	TMCC1
ANXA7	CSNK1G3	GGH	LRRTM1	PET117	RPS7	TMED5
API5	CSTF2T	GHR	LRRTM2	PEX13	RPSA	TMEM106A
ARF1	CTAGE5	GINM1	LRRTM3	PEX26	RSL24D1	TMEM106B
ARFGEF1	CTBP1	GJA1	LSM14A	PGK1	RTN4	TMEM117
ARFIP1	CTDSPL2	GLOD4	LYAR	PHKB	RWDD3	TMEM126B
ARGLU1	CTNNBIP1	GLRX5	LYPLAL1	PIANP	SAP18	TMEM156
ARHGAP12	CTSB	GMDS	LYRM9	PID1	SCAMP5	TMEM170B
ARHGAP5	CTSC	GMNN	LYZ	PITHD1	SCFD1	TMEM184C
ARL14EP	CWF19L1	GMPS	MAK16	PKNOX1	SCN1B	TMEM30A
ARL2BP	CXXC1	GNG7	MAP1B	PLAC8	SCYL2	TMEM47
ARL5B	CYP4V2	GNPDA2	MAP3K12	PLCB1	SDR42E1	TMOD1
ARRDC3	DBC1	GNPTG	MAPK10	PLS3	SEC11A	TMPO
ASF1A	DCLRE1C	GOLGA5	MAT2B	PNMA1	SEC23A	TMSB4X
ASTN1	DCP2	GORASP2	MCM4	PNPLA4	SEC62	TNKS
ATF4	DDT	GOSR1	MDM2	PNRC1	SEPP1	TOLLIP
ATG4C	DDX50	GPAM	MED13L	POLE3	SERPINB6	TOM1L1
ATP5J	DECR2	GPHN	MED23	POLE4	SERTAD2	TOMM70A
ATP6V0E1	DFFA	GPR1	MEF2A	POLR1D	SH2D5	TPBG
ATP8A1	DHRS3	GPR37	MGAT1	POLR2D	SHMT1	TPRG1L
ATPBD4	DICER1	GPR75	MLF1	POU3F4	SIKE1	TRAM1L1
AZI2	DNAJC1	GPR85	MMADHC	POU5F1	SIN3A	TRIB1
B4GALT4	DNAJC19	GRAMD3	MOBP	PPFIA2	SLC1A1	TRIM22
B4GALT7	DNAJC27	GRB10	MPHOSPH10	PPID	SLC1A3	TRMT5
BANF1	DNAJC3	GRID1	MPHOSPH6	PPM1B	SLC25A26	TSN
BCCIP	DRD1	GSKIP	MRP63	PPM1D	SLC25A3	TSPAN15
BEND5	DROSHA	GSTO1	MRPL10	PPP1R12A	SLC2A3	TSPAN5
BET1	DTWD1	GTDC1	MRPL41	PPP2R1A	SLC31A1	TTL
BIRC2	DTWD2	HADH	MRPS16	PRMT2	SLC35F1	TUFM
BRI3	DUSP22	HAX1	MRPS17	PRPSAP1	SLC39A10	TUSC3
BRIX1	DYRK2	HECTD1	MRPS28	PRRG1	SLC4A8	TWSG1
BTG3	EEF1B2	HEXA	MT3	PSMA2	SLC7A1	TXN
C1GALT1C1	EFNA5	HIATL1	MTA3	PSMA3	SLC8A1	TXNIP
CA2	EIF1AX	HIST1H2BK	MTAP	PSMB1	SLITRK1	TXNL1
CAAP1	EIF4A2	HNMT	MTDH	PSMD1	SLITRK2	TXNL4A
CACNB2	ELOVL4	HS3ST5	MTMR6	PSMG2	SMAD4	UAP1
CACNG3	EMC4	HSBP1	MYO6	PSPC1	SMYD3	UBE2E2
CARS	EMP2	HSPA13	MYT1L	PTEN	SNAPC3	UBE2E3
CCAR1	ENC1	HSPA5	NAB1	PTP4A2	SNCG	UBE2F
CCDC127	EP300	HTR2A	NANP	PVR	SNRK	UBE2M
CCDC90B	ERLIN2	HTRA1	NAP1L3	PXMP2	SNRPD3	UBE2V2
CCND3	EXOC2	HUWE1	NAPA	RAB18	SNRPN	UBE3A
CCT5	EXOC3	IAH1	NAPG	RAB1A	SNTA1	UBFD1
CD47	FAIM	ICA1	NBN	RAB5A	SP4	UCK1
CD9	FAM107A	IFNGR1	NCAPG2	RAB6A	SPATA2	UNC50
CD99L2	FAM127A	IGF1R	NDFIP1	RABAC1	SPIN3	URM1
CDC16	FAM134B	IKZF2	NDN	RABL2B	SPOPL	USP15
CDC5L	FAM136A	IL6ST	NDNL2	RABL5	SRSF7	USP46
CDC7	FAM168B	ILF2	NDUFV2	RALA	SS18	UST
CDH7	FAM174A	IMMP2L	NECAP2	RALYL	SSU72	UTP23
CDH8	FAM20B	IMP4	NEFL	RANBP6	ST3GAL1	VKORC1L1
CDK17	FAM20C	IMPACT	NEGR1	RAP1A	ST3GAL5	VMAC
CDO1	FAM49A	INPP4A	NFKB1	RARS	ST8SIA4	VMP1
CDR2	FAR2	INPP5K	NIPA2	RASGRP3	STAMBP	VPS26B
CECR5	FBXL3	IRF2	NKX6-2	RBFOX2	STARD13	VSTM2L
CEP19	FBXL4	IRGQ	NLGN1	RBPJ	STARD4	VTA1
CERK	FBXO3	JAK2	NOC4L	RFC5	STK24	VWA1
CETN3	FBXO30	JKAMP	NOL4	RGMA	STMN2	VWF
CHCHD2	FCAR	JUN	NOTCH2	RGS2	STX8	WBSCR17
CHMP4C	FCHSD2	KCNJ2	NPTX1	RGS5	SUCLG2	WDR12
CHORDC1	FDFT1	KCNQ5	NPY1R	RGS6	SUPT3H	WDR37
WSB1	ZC4H2	ZNF518B	YBEY	ZNF287	ZNF250	ZNF790
WSCD1	ZMYND11	ZNF596	YES1	ZNF32	XPOT	ZC3H7A
XPNPEP1	ZMYND19	ZNF671	ZBED4	ZNF345	ZNF770	ZNF501
XPO1	ZNF22					

Supplemental Table 1 (iv): H3K9me3 Imprinting Targets

H3K9me3-enriched Imprinting genes not detectable by RNA-Seq
DDIT4L
PTGFR

H3K9me3-enriched imprinting genes detectable by RNA-Seq
AGPAT5
JKAMP
LPHN3
MAPK10
NEGR1
NKX6-2
ST8SIA4
THAP1
WSCD1
